# Analysis of the genotype-phenotype correlation in patients with phenylketonuria in mainland China

**DOI:** 10.1038/s41598-018-29640-y

**Published:** 2018-07-26

**Authors:** Nana Li, Chunhua He, Jing Li, Jing Tao, Zhen Liu, Chunyan Zhang, Yuan Yuan, Hui Jiang, Jun Zhu, Ying Deng, Yixiong Guo, Qintong Li, Ping Yu, Yanping Wang

**Affiliations:** 1 National Center for Birth Defect Monitoring, West China Second University Hospital, Sichuan University, Chengdu, China; 2 Key Laboratory of Birth Defects and Related Diseases of Women and Children (Sichuan University), Ministry of Education, Chengdu, China; 3 Laboratory of Translational Medicine, Chinese PLA General Hospital, Beijing, China; 4 Tianjin Medical Laboratory, BGI-Tianjin, BGI-Shenzhen, Tianjin, 300308 China; 5 BGI-Shenzhen, Shenzhen, 518103 China; 6 China National GeneBank, BGI-Shenzhen, Shenzhen, 518120 China

## Abstract

Mutations in the gene encoding phenylalanine hydroxylase (PAH) are associated with various degrees of phenylketonuria (PKU). The aim of our study was to define the genotype-phenotype correlations of mutations in the PAH gene that cause phenylketonuria (PKU) among the Chinese mainland population. Mutations in the PAH gene were analysed by next-generation sequencing, and a genotype-phenotype correlation analysis was performed in 1079 patients. Fifteen “null + null” genotypes, including four homoallelic and eleven heteroallelic genotypes, were clearly associated with classic PKU. Five functionally hemizygous (p.E280K, p.R252Q, p.E56D, p.S310F and p.T372R) and four compound heterozygous (p.T278I/p.S359L, p.R408W/p.R243Q, p.F161S/p.R243Q and p.F161S/p.R413P) genotypes were clearly associated with classic PKU. Ten functionally hemizygous genotypes, p.G257V, p.R158W, p.L255S, p.G247V, p.F161S, p.R158Q, p.V388M, p.I65T, p.I324N and p.R400K, were frequently associated with classic PKU. Three functionally hemizygous genotypes, p.P147L, p.I95del and p.F331S, and four compound heterozygous genotypes, p.G257V/p.R408Q, p.A434D/p.R413P, p.R243Q/p.A47E and p.R241C/p.G239D, were consistently correlated with mild PKU. Three functionally hemizygous genotypes, p.H107R, p.Q419R and p.F392I, and nine compound heterozygous genotypes (p.G312V/p.R241C, p.R243Q/p.V230I, p.R243Q/p.A403V, p.R243Q/p.Q419R, p.R243Q/p.R53H, p.R243Q/p.H107R, p.R241C/p.R408Q, p.R241C/p.H220P and p.R53H/p.R400K) were consistent with mild hyperphenylalaninaemia (MHP). Our study provides further support for the hypothesis that the PAH genotype is the main factor that determines the phenotype of PKU.

## Introduction

Phenylketonuria (PKU, OMIM #261600) is an inborn error in phenylalanine (Phe) metabolism, with an autosomal recessive mode of inheritance. The severity of the disorder varies between patients and is classified as mild hyperphenylalaninaemia (MHP), mild PKU (mPKU), and classic PKU (cPKU), depending on the blood Phe level at the time of diagnosis or dietary Phe tolerance^1^. The prevalence of PKU is approximately 1 in 15,000 individuals, but differs among different populations^2^. In mainland China, the average incidence is 1 in 11614^3^, and in Taiwan, the average incidence is 1 in 55057^4^.

The gene causing PKU in patients is phenylalanine hydroxylase (PAH), which is located on chromosome 12 (region 12q22-q24.2). The PAH gene (Gene ID: 5053) spans approximately 90 kb, consisting of 13 exons and 12 large introns. The full-length PAH cDNA encodes a protein with a molecular weight of approximately 52 kDa (452 AAs) that is assembled as a homotetramer in the mature form. Each monomer consists of three functional domains: an N-terminal regulatory domain (residues 1–142); a catalytic domain (residues143–410) that includes binding sites for the Fe^3+^ ion, which is reduced to the active Fe^2+^ form upon the binding of a cofactor; and a C-terminal oligomerization domain (residues 411–452) with dimerization (residues 411–426) and tetramerization motifs (residues 427–452). To date, more than 900 different mutations have been identified in PAH and recorded in the locus-specific database (LSD) PAHvdb (http://www.biopku.org/pah/). These mutations are scattered throughout the PAH gene. Depending on the mutation type and position, the effects of a mutation on the structure and activity of the PAH vary substantially. Consequently, the activity of the mutant protein ranges from 0% to approximately 100% compared to the normal PAH enzyme^5^. Correspondingly, phenylketonuria phenotypes range from mild hyperphenylalaninaemia (MHP) that does not require treatment to classic PKU, which is characterized by severe mental retardation and epilepsy in the absence of treatment.

Accumulating knowledge of the PAH gene has enabled research to propose a strong correlation between mutations and various metabolic states. The wide range of metabolic phenotype is mainly determined by the PAH genotype^6,7^, although other factors might also exert effects^8^. Information provided by sequencing patients’ alleles enables researchers to not only predict the severity of the disease but also provide the physician with an effective prognostic interpretive tool to establish a better tailored diet^9^. For more than two decades, efforts have been devoted to obtaining a complete understanding of the effects of mutations on the PKU phenotype. Based on the predicted residual activity (PRA) derived from *in vitro* expression data^10,11^ or the sums of assigned phenotypic effect of mutant PAH alleles (AV scores) derived from a more formalized system developed by Guldberg^7^, several studies have been performed to establish the degree of genotype-phenotype correlation in European and Chinese populations and have revealed clear associations between some mutations and the severity of disease^12–19^. Due to the large number of mutations and the low population frequency of some of these mutations, the phenotypic consequences of a given mutation are often difficult to ascertain, and correlation analyses may also give rise to conflicting results^12^. Recently, one study using data from the up-to-date LSD PAHvdb and genotype database BIOPKU showed that enzyme stability algorithms (FoldX andSNPs3D), allelic phenotypes and enzyme activities were the most powerful predictors of patients’ phenotypes^20^.

In our previous study, we reported a spectrum of PAH mutations complied from a large cohort of 796 patients with PKU in mainland China and identified 194 mutations^21^. Furthermore, we assessed the correlation between genotype and the tetrahydrobiopterin-responsive phenotype, and identified mutations responsive to the tetrahydrobiopterin (BH4) treatment^22^. In the present study, we performed an analysis of the genotype-phenotype correlations for 534 different genotypes in 1079 Chinese patients (the genetic analysis of 682 patients was conducted in our previous study).

## Results

### Classification of patient phenotypes

**Figure 1:**
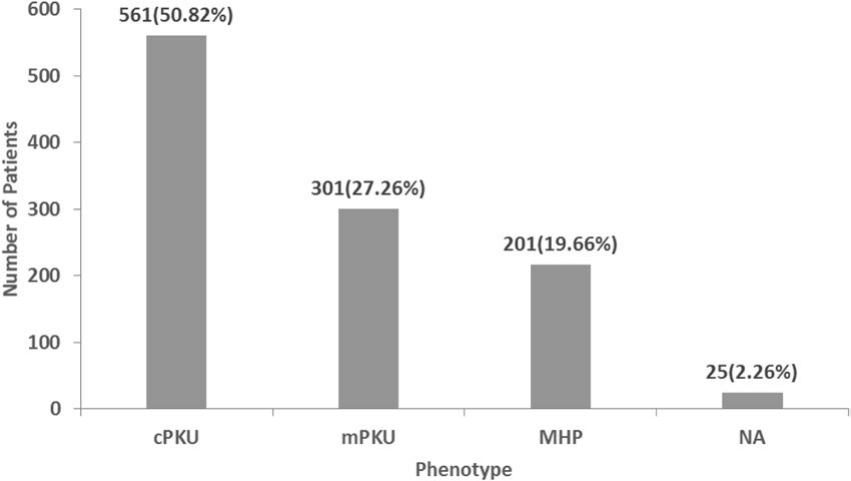
Classification of patient phenotypes.

Three phenotypes were classified according to the pretreatment plasma phenylalanine levels: classic PKU, mild PKU and MHP^1^. The classification of patient phenotypes is shown in Fig. 1, among the 1104 patients, 217 were classified as having MHP (17.63%), 301 as having mPKU (26.75%), and 561 as having cPKU (53.38%); information about the clinical phenotype of the remaining 26 patients (2.25%) was not available.

### Mutation spectrum

A spectrum of 203 different mutations was identified in the 1104 patients, including132 missense mutations (65.02%), 28 splice-site mutations (13.79%), 20 deletions (9.85%), 18 nonsense mutations (8.87%), 4 insertions (1.97%), and 1 silent mutation (0.49%). The mutations were distributed throughout the entire gene, with the exception of intron 9.

In terms of the mutation frequency, p.R243Q was the most prevalent mutation, with a relative frequency of 20.1993%. In addition, seven mutations, p.EX6-96A > G, p.V399V, p.R241C, p.R111*, p.R413P, p.Y356*, and IVS4-1G >A, were detected at at relatively high frequencies (7.8351%, 6.2953%, 5.9783%, 5.163%, 5.163%, 4.846%, and 4.0761%, respectively) (Table S1). 1 novel mutations were identified: p.E228G.

**Figure 2:**
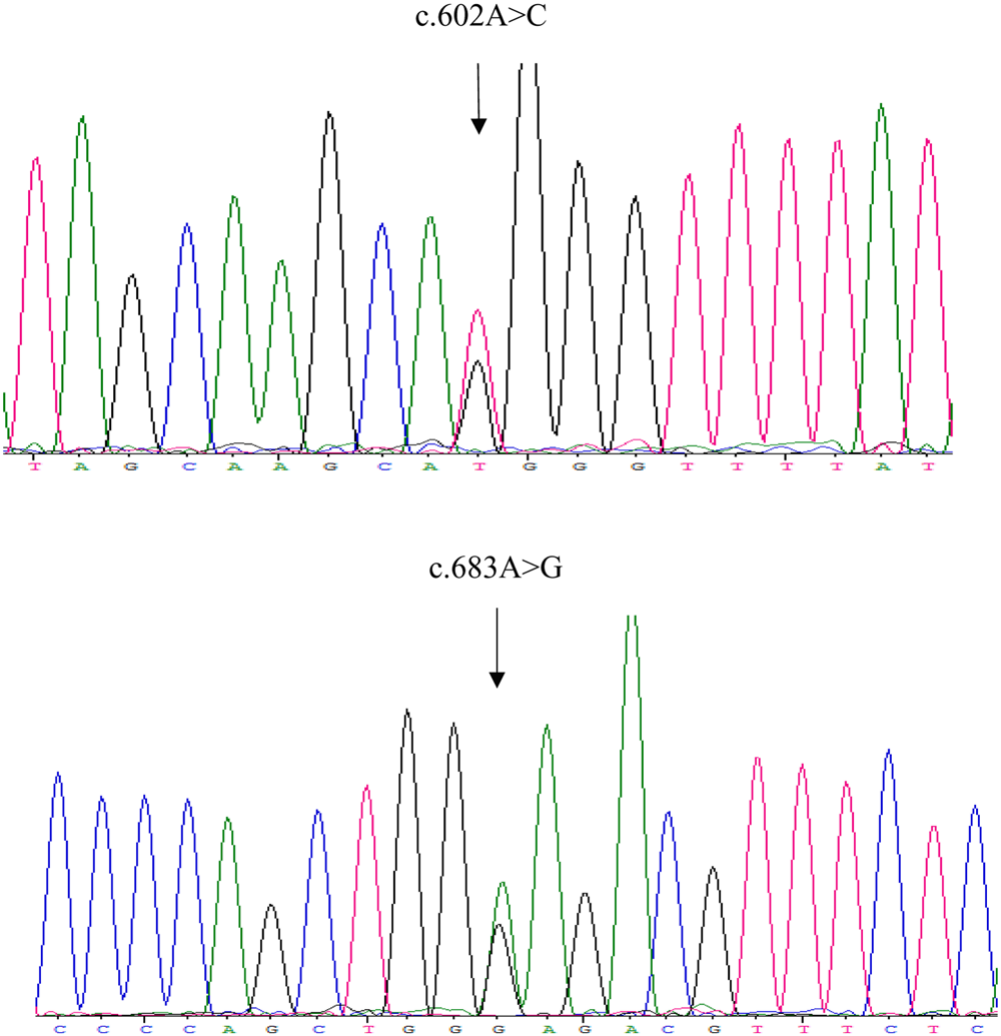
Two novel mutations, c.602A > C and c.683A > G.

Two novel mutations were identified: p.H201P (c.602A > C) and p.E228G (c.683A > G). The chromatograms obtained from Sanger sequencing are shown in Fig. 2.

### Genotype-phenotype correlations

The 203 different PAH mutations were combined into 534 different genotypes, including homozygous (n = 26) and compound heterozygous genotypes (n = 508), and are listed in Table S2. The compound heterozygous genotype was divided into null + null mutations (n = 83), null+ missense mutations (n = 248) and missense + missense mutations (n = 177). Nine hundred eighty-seven patients exhibited the compound heterozygous genotype (89.40%), while the remaining 117 patients displayed the homozygous genotype (10.60%). A genotype-phenotype correlation study was performed on the 1079 patients for whom clinical information was available.

### Homoallelic mutant PAH genotypes

Among the 26 homozygous genotypes, 12 genotypes were present in more than one patient. Four homoallelic mutant genotypes, p.Y166*, p.Y325*, p.Y356*, p.V399V, were associated with cPKU in 100% of patients, the numbers of patients harbouring these genotypes were three, two, six and four, respectively. The homoallelic mutant genotype p.S70del was associated with mPKU in 100% of patients and was detected in two patients. The homoallelic mutant genotype p.R241C was associated with MHP in 100% of patients, and the genotype was detected in two patients. Three homoallelic mutant genotypes, c.441 + 3G > C, p.R413P and p.S349A, conferred two phenotypes. Among the patients displaying the 2 c.441 + 3G > C homozygous genotype, one had cPKU and one had mPKU. Four of the five patients with the p.R413P homozygous genotype had cPKU, and the remaining patient had mPKU. Among the 2 patients with the p.S349A homozygous genotype, one had MHP and the other had mPKU. Six patients with the p.R111* homozygous genotype, eight patients with the p.EX6-96A > G homozygous genotype and fifty-eight patients with the p.R243Q homozygous genotype were categorized into all three phenotype categories. Each of the remaining 14 genotypes was only present in one patient.

### “Null + null” heteroallelic PAH genotypes

Among the 167 patients harbouring “null + null” heteroallelic PAH genotypes, 117 had cPKU, 31 had mPKU, and 17 had MHP. Among the 26 genotypes that were present in more than one patient, 13 genotypes showed consistent phenotypes in all patients. Eleven genotypes were associated with cPKU, including p.R111*/p.Y356*, p.R111*/C.442-1G > A, C.442-1G > A/C.441 + 3G > C, C.442-1G > A/p.R176*, C.442-1G > A/p.V399V, C.442-1G > A/p.EX6-96A > G, C.442-1G > A/p.Q232*, p.Y166*/p.EX6-96A > G, p.R176*/p.Y356*, p.R176*/p.EX6-96A > G and p.EX6-96A > G/p.V399V. The p.S70del/p.EX6-96A > G genotype was correlated with mPKU. The p.V399V/C.1315 + 6T > A genotype correlated with MHP. Seven genotypes, p.S70del/p.R111*, p.S70del/p.V399V, p.S70del/p.Y356*, p.R111*/p.V399V, p.EX6-96A > G/p.R241Pfs*100, p.R252W/p.V399V and p.Y356*/p.V399V, were associated with cPKU and mPKU. Two genotypes, p.EX6-96A > G/C.1315 + 6T > A and p.R261*/p.V399V, were correlated with MHP and mPKU. The p.EX6-96A > G/p.Y356* genotype correlated with MHP and cPKU. Three genotypes, p.S16*/p.V399V, p.R111*/p.EX6-96A > G and C.442-G > A/p.Y356*, were associated with all three phenotypes. Of the remaining 57 genotypes, 38 genotypes were associated with cPKU, 13 genotypes were associated with mPKU, and 6 genotypes correlated with MHP.

### Functionally hemizygous genotypes

Two hundred forty-eight genotypes were formed by 89 different missense mutations combined with null mutations. An assessment of missense mutations combined with null mutations would facilitate the determination of the intrinsic severity of those missense mutations. Thirty-one genotypes, including 11 missense mutations, showed consistent phenotypes in all patients. The p.E280K, p.R252Q, p.E56D, p.S310F and p.T372R mutations were associated with cPKU and the p.P147L, p.I95del and p.F331S mutations were associated with mPKU, while the p.Q419R, p. H107R and p.F392I mutations were associated with MHP. Although 12 mutations, including p.G257V, p.R158W, p.L255S, p.G247V, p.F161S, p.R158Q, p.A342T, p.V388M, p.I65T, p.I324N, p.R400K, and p.L430P, were observed in patients with mPKU and cPKU, except for p.A342T and p.L430P, most of the remaining 10 mutations were frequently detected in patients with cPKU. Three mutations, p.R241H, p.L308F, and p.T418P, were identified in patients with MHP or mPKU. Two mutations, p.R408W and p.A345T, were detected in patients with MHP or cPKU. Ten functionally hemizygous genotypes, including p.A434D, p.R243Q, p.R241C, p.R413P, p.R261Q, p.R408Q, p.R53H, p.G247R p.M276K and p.S349A, appeared in patients with the three phenotypes, but specific genotypes, including p.G247V/p.V399V, p.R243Q/p.Y166*, p.R243Q/p.Y176*, p.R413P/p.Y356*, p.R413P/C.442-1G > A, were only detected in patients with cPKU. The remaining 50 mutations were only identified in patients in one phenotype category.

### Compound heterozygous genotypes

One hundred seventy-seven different compound heterozygous genotypes were harboured by 321 individuals. Among the 40 genotypes that were present in more than one patient, 17 genotypes showed consistent phenotypes in all patients. The p.T278I/p.S359L, p.R408W/p.R243Q, p.F161S/p.R243Q and p.F161S/p.R413P genotypes were correlated with cPKU; the p.G257V/p.R408Q, p.A434D/p.R413P, p.R243Q/p.A47E and p.R241C/p.G239D genotypes were correlated with mPKU; and the p.G312V/p.R241C, p.R243Q/p.V230I, p.R243Q/p.A403V, p.R243Q/p.Q419R, p.R243Q/p.R53H, p.R243Q/p.H107R, p.R241C/p.R408Q, p.R241C/p.H220P and p.R53H/p.R400K genotypes were correlated with MHP. Thirteen genotypes (p.P281L/p.R243Q, p.R252Q/p.R243Q, p.G247V/p.R243Q, p.R243Q/p.V388M, p.R243Q/p.I65T, p.R243Q/p.R413P, p.R243Q/p.G247R, p.R243Q/p.I324N, p.R243Q/p.A345T, p.R243Q/p.S349A, p.R243Q/p.R400T, p.R241C/p.I65T and p.R413P/p.R408Q) were detected in patients with mPKU and cPKU. Four genotypes, p.A434D/p.R261Q, p.R241C/p.A156P, p.R241C/p.S349A and p.V388M/p.I421T, were identified in patients with MHP and mPKU. Six genotypes, p.A434D/p.R243Q, p.R243Q/p.R241H, p.R243Q/p.R241C, p.R243Q/p.R261Q, p.R243Q/p.R408Q and p.R241C/p.R413P, appeared in the three phenotype categories. The remaining 137 genotypes were only observed in one phenotype category.

## Discussion

In this study, we first described the mutation spectrum of the PAH gene in 1104 patients with phenylketonuria, and then examined genotype-phenotype correlations.

Among the 203 mutations identified in our study, 33 mutations were only detected in the patients enrolled in the present study, 170 mutations were consistent with the mutations reported in our previous articles^21^. Among the 170 mutations, 102 mutations were detected only in the 682 patients enrolled in our previous study, whereas 68 mutations were detected in both newly enrolled patients and previously examined patients. The prevalent mutations, as assessed by the relative frequency, included p.R243Q, p.EX6-96A > G, p.V399V, p.R241C, p.R111*, p.R413P, p.Y356*, and c.442-1G > A, consistent with a previous study of a Chinese mainland population^21^.

Genotype-phenotype correlation analyses are the cornerstone of most studies on metabolic diseases. PKU mutations detected in Chinese patients are highly heterogeneous. In our study, the genotypes of most patients were heterozygotes (89.4%); classic PKU comprised the predominant type found in our samples (50.82%) compared with mild PKU and MHP (27.26% and 19.66%, respectively). Studies of the genotype-phenotype correlations between homoallelic mutant PAH genotypes and null + null and null + missense (functionally heterozygous) genotypes enabled us to discover the effect of a single mutation on the phenotype^23^.

Mutations that cause the severe (classic) phenotypes are those in which the PRA is less than 10%^24^. Overall, a good genotype-phenotype correlation was observed for patients carrying null mutations in both alleles including homozygous. In our samples, 29 of 37 patients carrying homoallelic genotypes and 117 of 167 patients carrying heteroallelic genotypes were classified as having cPKU, showing the highest degree of concordance with the most severe phenotypes^13,17^. Four homoallelic mutant genotypes, p.Y166*, p.Y325*, p.Y356*, and p.V399V, and 11 heteroallelic genotypes, p.R111*/p.Y356*, p.R111*/C.442-1G > A, C.442-1G > A/C.441 + 3 G > C, C.442-1G > A/p.R176*, C.442-1G > A/p.V399V, C.442-1G > A/p.EX6-96A > G, C.442-1G > A/p.Q232*, p.Y166*/p.EX6-96A > G, p.R176*/p.Y356*, p.R176*/p.EX6-96A > G and p.EX6-96A > G/p.V399V, were clearly associated with classic PKU, as all the patients harbouring these genotypes were classified as having cPKU. The p.E280K mutation and p.R252Q mutation, which were both predicted to be deleterious, had PRAs of 2% and 3%, respectively, indicating that these two mutations were associated with the severe phenotype. In our sample, 6 patients harbouring the p.E280K hemizygous mutation and 8 patients carrying the p.R252Q hemizygous mutation displayed the cPKU phenotype, showing concordance between *in vitro* and *in vivo* phenotypes. Notably, the p.G257V, p.R158W, p.L255S, p.G247V and p.F161S genotypes, all of which were predicted to be deleterious, showed 1%, 2%, 2%, 4%, and 7% PRAs, respectively. Although patients carrying these mutations in their functionally hemizygous state displayed mPKU and cPKU phenotypes, the majority exhibited cPKU, suggesting that these mutations were frequently correlated with cPKU.

Potentially “functional hemizygous genotypes” may help researchers predict residual PAH activity due to specific pathogenic variants, if the null mutations have limited residual PAH activity. The influence of the PAH activity of seven missense mutations, p.E56D, p.H107R, p.S310F, p.I324N, p.P147L, p.F331S and p.R400K, has not been determined. In our study, patients carrying the functionally hemizygous p.E56D genotype, which is predicted to be tolerated, and the functionally hemizygous p.S310F genotype, which is predicted to be deleterious, were all classified as displaying cPKU, suggesting that these genotypes correlated with cPKU. Moreover, these two mutations might result in less than 10% enzyme activity. This finding was consistent with the results from a previous study showing that the hemizygous p.S310F mutation is associated with cPKU in a cohort of Syrian patients with PKU^18^. The p.P147L and p.F331S genotypes, which were both predicted to be deleterious, were associated with mPKU in their functionally hemizygous state, suggesting that these two mutations resulted in greater residual enzyme activity. In addition, the p.H107R mutation that was predicted to be tolerated was correlated with MHP, as both patients carrying this mutation in its functionally hemizygous state showed the MHP phenotype, suggesting that these two mutations resulted in greater residual enzyme activity. The p.I324N mutation, which was predicted to be deleterious, and the p.R400K mutation, which was predicted to be tolerated, appeared to be associated with cPKU because the majority of patients (3/4 and 4/5, respectively) carrying the functionally hemizygous genotypes displayed cPKU, suggesting that these two mutations might result in less than 10% enzyme activity.

According to previous studies^5,7,25^, “disease severity in most cases is determined by the least severe of two PAH mutations.” Thus, when one of the mutations exerts a severe effect and the second one allows for at least a partially functioning PAH allele, the HPA metabolic phenotype will be less severe. As expected, the p.Q419R mutant for which the extent of damage was not able to be predicted has a PRA of 71%, and in our study, ten patients bearing the p.Q419R/null genotype all presented MHP. Similarly, the p.R241C and p.R408Q mutations, which were both predicted to be deleterious, showed PRAs of 25% and 46%, respectively; four patients with the p.R241C/p.R408Q genotype had MHP. The same trend was observed for the genotypes p.R243Q/p.V230I (14% and 63% PRA, respectively) and p.R243Q/p.R53H (14% and 79% PRA, respectively). In our study, these two genotypes were correlated with MHP in 4 and 6 patients, respectively. In addition, p.I95del exhibited 27% PRA, and both patients carrying the functionally hemizygous genotype exhibited the mPKU phenotype. The residual PAH activity that was predicted from “functional hemizygous genotypes” can be used to analyse functional effects of compound heterozygous genotypes if appropriate alleles are present. The p.R243Q mutation has a PRA of 14%, the p.H107R is predicted to exhibit greater residual enzyme activity, and the compound heterozygous p.R243Q/p.H107R genotype was predicted to be associated with MHP. This finding was consistent with the observation that both patients carrying p.R243Q/p.H107R presented MHP in thr present study. The p.R53H mutation has a PRA of 79%, the p.R400K is predicted to display less than 10% residual enzyme activity, and the compound heterozygous p.R53H/p.R400K mutation was presumed to be associated with MHP. This finding was consistent with the results obtained from both patients carrying the p.R243Q/p.H107R genotype in the present study.

Notably, a number of mutations that showed substantial *in vitro* activities resulted in severe clinical phenotypes. As an example, the p.V388M in the PAH gene that was predicted to be deleterious showed a PRA of 28%, and four of five patients harbouring the p.V388M/null genotype displayed the cPKU phenotype. The result was consistent with the results that patients with PKU in Japan and Korea who carry the functionally hemizygous p.V388M mutation had cPKU^14,19^, but was inconsistent with previous study conducted in China in which patients bearing genotypes composed of p.V388M and any known null allele exhibited cPKU and mPKU^16^. Likewise, the p.R243Q mutant that was predicted to be deleterious showed a PRA of 14%. However, this mutation appeared to be associated with a severe phenotype, as deduced by the observation that most patients (106/156) carrying the functionally hemizygous genotype displayed cPKU and four patients carrying the compound heterozygous p.R243Q/p.R408W genotype were all classified as having cPKU, consistent with a previous report^26^. In addition, p.R158Q, p.I65T and p.R413P, all of which were predicted to be deleterious, showed 10%, 33% and 35% PRA, respectively. The majority of the patients carrying these mutations in functionally hemizygous state, as well as three of four patients carrying p.R158Q, six of seven patients carrying p.I65T and thirty-four of forty-six patients carrying p.R413P, displayed cPKU. Interestingly, both patients carrying p.R243Q/p.I65T and the majority patients (24/29) of carrying p.R243Q/p. R413P displayed cPKU.

In our study, discordance was observed between *in vitro* and *in vivo* phenotypes. For example, the PAH enzymatic activity of the p.A434D mutation was 3%, and the extent of damage was predicted to be deleterious. However, patients carrying its functionally hemizygous genotypes showed all three phenotypes, but only a small percentage (1/17) had cPKU. In addition, the PAH enzymatic activity of the p.R413P mutations was 35%, and the extent of damage was predicted to be deleterious. However, the patients carrying its functionally hemizygous genotypes showed all three phenotypes; furthermore, the majority (34/46) had cPKU.

Importantly, some patients with the same genotype had different phenotypes. Among the most frequent mutations detected in Chinese patients, the homozygous p.R243Q mutation produced all three phenotypes: 2 patients with MHP, 5 with mPKU, and 27 with cPKU. In one Chinese study^27^, nine patients carrying this genotype showed cPKU, but in another study^16^, 22 patients showed other phenotypes, with the exception of MHP. These results were inconsistent. The p.R413P mutation produced similar results. Three patients with the homozygous p.R413P genotype were classified as having cPKU, and one as having mPKU, findings that are inconsistent with a previous study conducted in China in which three patients were diagnosed with cPKU^16^. Likewise, homozygous p.EX6-96A > G and p.R111* mutations also produced inconsistent phenotypes, and yielded contradictory findings compared with a previous study^27^. In addition, 13 compound heterozygous genotypes were detected in patients with mPKU and cPKU. Four genotypes were identified in patients with MHP and mPKU, and 6 genotypes appeared in the three phenotype categories.

A theory that attempts to explain the variations in plasma Phe concentrations in individuals with the same genotypes^8,28,29^ suggests that some missense mutations affect protein folding, thus altering the oligomerization of the nascent PAH protein. This process is likely influenced by an individual’s genetic background, including potential differences in the quality and quantity of chaperones and proteases. In compound heterozygotes, the inconsistency could be explained by interallelic complementation between different subunits of heterotetrameric PAH^30,31^. Additionally, since we noticed inconsistencies in patients with identical genotypes on the same population background, variations in modifier genes might explain interindividual inconsistencies, rather than interpopulation inconsistencies^23^. In homozygotes, interallelic complementation does not explain the different serum Phe levels observed in some patients^32^. This phenomenon will probably be explained in the future by the identification of new transcriptional regulators located in the non-coding region of the PAH gene and/or a variety of modifier genes. In addition to genetic influences on the genome, the identification of a number of epigenetic and/or environmental modifiers associated with PKU would lay the framework for an improved understanding of the nuances of the disease course and treatment response^33^.

By performing an analysis of a larger sample size, our study provided further evidence supporting the hypothesis that the wide range of PKU phenotypes is mainly determined by different mutations within the PAH gene^7,25^. Notable results include the identification of clear correlations between fifteen “null + null” genotypes and classic PKU. Four hemizygous and four compound heterozygous mutations in the PAH gene were precisely correlated with classic PKU. Ten hemizygous mutations in the PAH gene were associated with classic PKU. Three functionally hemizygous genotypes and four compound heterozygous genotypes were consistently correlated with mild PKU. Two functionally hemizygous genotypes and nine compound heterozygous genotypes were associated with MHP. The results from this study provide very valuable insights that will enable predictions of patients’ clinical presentation. However, our study also revealed substantial discordance between the PAH genotype and phenotype in a Chinese population. Further studies are needed to understand genotype-phenotype correlations and elucidate inconsistencies.

## Materials and Methods

### Subjects

One thousand one hundred four unrelated patients carrying two mutations were enrolled from 29 separate newborn screening centres in China. For more information about the inclusion and enrolment of patients, and questionnaire information, please refer to our previously published articles^21^. TParental permissions and informed consents were obtained from the parents of all patients. The study was approved by the Ethics Committee of West China Second University Hospital, Sichuan University (No: 2015011) and adhered to the tenets of the Declaration of Helsinki.

Patients were assigned to one of three separate phenotype categories according to their pretreatment plasma Phe levels, including mild hyperphenylalaninaemia (MHP, Phe 120–600 μmol/L), mild PKU (mPKU, Phe 600–1200 μmol/L), and classic PKU (cPKU, Phe >1200 μmol/L), together with a group of patients whose phenotypes were unavailable.

### Genotype analysis

For a detailed description of methods used to collect blood samples and extract DNA, please refer to our previously published article^21^. All 13 exons and their surrounding introns of the PAH gene, which covered 200 bp upstream and 200 bp downstream of the exons, were sequenced using next-generation sequencing (Shenzhen, Guangdong province, China). All mutations were described as reference sequences (NM_000277.2, NP_000268.1). The extent of damage caused by each PAH mutation was predicted based on the SIFT value and SIFT interpretation in the Biopku database (http://www.biopku.org/pah/). The validation tests on parents were performed using Sanger sequencing. For a detailed description of the experimental procedures, please refer to our previous article^21^.

### Genotype-phenotype analysis

In our analysis, predicted residual PAH activity (PRA) was assessed for each mutation according to data listed in the PAHvdb database (www.biopku.org/pah). This value was calculated as the average of the data obtained from eukaryotic expression systems. Nonsense and frame-shift variations, as well as missense mutations that result in zero enzyme activity *in vitro* (for example p.R252W), were defined as null. Splice-site variants affecting invariable ag and gt nucleotides were also considered null mutations, while splice-site variants in non-canonical sequences were defined as only putative-null mutations since they can produce a wild-type protein in some cases.

Genotypes were first divided into two categories: homoallelic mutant PAH genotypes and heteroallelic mutant PAH genotypes. Next, the heteroallelic PAH genotypes were further listed in approximately the order of increasing predicted residual activity (PRA), showing a transition from null + null through null + missense (functionally hemizygous) and finally to missense + missense (compound heterozygous) mutations.

## Electronic supplementary material

Supplementary material
