# Supplementary material for: Analysis of the genotype-phenotype correlation in patients with phenylketonuria in mainland China
Source: Sci Rep. 2018 Jul 26;8:11251. doi: 10.1038/s41598-018-29640-y (PMC6062512; doi:10.1038/s41598-018-29640-y)
Supplement: Supplementary file 1 — Supplementary material [file 41598_2018_29640_MOESM1_ESM.docx]

**Analysis of the genotype-phenotype correlation in patients with phenylketonuria in mainland China**

Nana Li^1,2*^, Chunhua He^1,2^*, Jing Li^3*^ , Jing Tao^1,2^, Zhen Liu^1,2^, Chunyan Zhang^3^, Yuan Yuan^4^,Hui Jiang ^5,6^, Jun Zhu^1,2^, Ying Deng^1,2^, Yixiong Guo^1,2^, Qintong Li^2^, Ping Yu^1,2^ & Yanping Wang ^1,2^

Table S1. The spectrum of PAH mutations

| Systematic name | Trivial name | Type of mutation | Location | Domain | No.of alleles | | | | RF(%) |
| --- | --- | --- | --- | --- | --- | --- | --- | --- | --- |
|  |  |  |  |  | MHP | mPKU | cPKU | NA |  |
| c.3G>C | p.M1I | missense | E1 | regulatory | 0 | 1 | 0 | 0 | 0.0453 |
| c.13delG | p.V5Sfs*33 | deletion | E1 | regulatory | 0 | 0 | 1 | 0 | 0.0453 |
| c.30_31insC | p.R13Qfs*5 | insertion | E1 | regulatory | 0 | 0 | 1 | 0 | 0.0453 |
| c.44_45delTC | p.S16* | deletion | E1 | regulatory | 0 | 1 | 1 | 0 | 0.0906 |
| c.47_48delCT | p.S16* | deletion | E1 | regulatory | 2 | 2 | 2 | 0 | 0.2717 |
| c.60+5_+6delGC |  | deletion | I1 | IVS | 0 | 0 | 1 | 0 | 0.0453 |
| c.113_115delTCT | p.F39del | deletion | E2 | regulatory | 0 | 0 | 3 | 0 | 0.1359 |
| c.116_118delTCT | p.F39del | deletion | E2 | regulatory | 0 | 0 | 2 | 0 | 0.0906 |
| c.124_126delAAA | p.K42del | deletion | E2 | regulatory | 0 | 0 | 1 | 0 | 0.0453 |
| c.127G>T | p.E43* | nonsense | E2 | regulatory | 0 | 0 | 2 | 0 | 0.0906 |
| c.140C>A | p.A47E | missense | E2 | regulatory | 0 | 3 | 0 | 0 | 0.1359 |
| c.143T>C | p.L48S | missense | E2 | regulatory | 0 | 1 | 0 | 0 | 0.0453 |
| c.155T>C | p.L52S | missense | E2 | regulatory | 1 | 0 | 0 | 0 | 0.0453 |
| c.158G>A | p.R53H | missense | E2 | regulatory | 14 | 4 | 4 | 2 | 1.0870 |
| c.168+5G>C |  | splice | I2 | IVS | 1 | 1 | 2 | 0 | 0.1812 |
| c.168G>T | p.E56D | missense | E2 | regulatory | 0 | 2 | 6 | 0 | 0.3623 |
| c.169+19T>C |  | splice | I2 | IVS | 0 | 1 | 0 | 0 | 0.0453 |
| c.184_187insCTGA | p.L62Pfs*7 | insertion | E3 | regulatory | 0 | 0 | 1 | 0 | 0.0453 |
| c.185_188dupTGAC | p.H64Dfs*4 | insertion | E3 | regulatory | 0 | 0 | 2 | 0 | 0.0906 |
| c.193A>G | p.I65V | missense | E3 | regulatory | 1 | 0 | 0 | 0 | 0.0453 |
| c.194T>C | p.I65T | missense | E3 | regulatory | 0 | 4 | 11 | 1 | 0.7246 |
| c.206_208delCTT | p.S70del | deletion | E2 | regulatory | 3 | 15 | 6 | 0 | 1.0870 |
| c.208_210delTCT | p.S70del | deletion | E2 | regulatory | 2 | 13 | 5 | 0 | 0.9058 |
| c.224A>T | p.D75V | missense | E3 | regulatory | 0 | 0 | 1 | 0 | 0.0453 |
| c.279_281delCAT | p.I95del | deletion | E3 | regulatory | 0 | 1 | 2 | 0 | 0.1359 |
| c.284_286delTCA | p.I95del | deletion | E3 | regulatory | 0 | 3 | 0 | 0 | 0.1359 |
| c.292T>G | p.L98V | missense | E3 | regulatory | 0 | 1 | 2 | 0 | 0.1359 |
| c.301G>A | p.D101N | missense | E3 | regulatory | 1 | 0 | 0 | 0 | 0.0453 |
| c.320A>G | p.H107R | missense | E3 | regulatory | 5 | 0 | 0 | 0 | 0.2264 |
| c.331C>T | p.R111* | nonsense | E3 | regulatory | 16 | 24 | 70 | 4 | 5.1630 |
| c.353-2A>G |  | splice | I3 | IVS | 1 | 0 | 0 | 0 | 0.0453 |
| c.353-2A>T |  | splice | I3 | IVS | 0 | 0 | 2 | 0 | 0.0906 |
| c.361T>G | p.F121V | missense | E4 | regulatory | 0 | 1 | 0 | 0 | 0.0453 |
| c.440C>T | p.P147L | missense | E4 | catalytic | 0 | 3 | 0 | 0 | 0.1359 |
| c.441+1G>A |  | splice | I4 | IVS | 0 | 0 | 1 | 0 | 0.0453 |
| c.441+2T>A |  | splice | I4 | IVS | 1 | 0 | 1 | 0 | 0.0906 |
| c.441+3G>C |  | splice | I4 | IVS | 0 | 5 | 6 | 0 | 0.4982 |
| c.442-1G>A |  | splice | I4 | IVS | 10 | 18 | 62 | 0 | 4.0761 |
| c.442G>C | p.G148R | missense | E5 | catalytic | 0 | 1 | 0 | 0 | 0.0453 |
| c.443G>T | p.G148V | missense | E5 | catalytic | 1 | 0 | 0 | 0 | 0.0453 |
| c.460T>C | p.Y154H | missense | E5 | catalytic | 1 | 0 | 1 | 0 | 0.0906 |
| c.464G>A | p.R155H | missense | E5 | catalytic | 1 | 0 | 0 | 0 | 0.0453 |
| c.466G>C | p.A156P | missense | E5 | catalytic | 1 | 2 | 6 | 0 | 0.4076 |
| c.470G>A | p.R157K | missense | E5 | catalytic | 0 | 0 | 1 | 0 | 0.0453 |
| c.472C>T | p.R158W | missense | E5 | catalytic | 0 | 4 | 4 | 0 | 0.3623 |
| c.473G>A | p.R158Q | missense | E5 | catalytic | 1 | 3 | 4 | 0 | 0.3623 |
| c.478C>T | p.Q160* | nonsense | E5 | catalytic | 1 | 0 | 0 | 0 | 0.0453 |
| c.482T>C | p.F161S | missense | E5 | catalytic | 1 | 1 | 15 | 0 | 0.7699 |
| c.494C>A | p.A165D | missense | E5 | catalytic | 0 | 0 | 1 | 0 | 0.0453 |
| c.498C>G | p.Y166* | nonsense | E5 | catalytic | 2 | 3 | 24 | 1 | 1.3587 |
| c.505C>A | p.R169S | missense | E5 | catalytic | 3 | 0 | 0 | 0 | 0.1359 |
| c.505C>G | p.R169G | missense | E5 | catalytic | 1 | 1 | 0 | 0 | 0.0906 |
| c.505C>T | p.R169C | missense | E5 | catalytic | 2 | 0 | 0 | 0 | 0.0906 |
| c.509+1G>A |  | splice | I5 | IVS | 0 | 0 | 1 | 0 | 0.0453 |
| c.509A>C | p.H170P | missense | E5 | catalytic | 1 | 0 | 0 | 0 | 0.0453 |
| c.509A>G | p.H170R | missense | E5 | catalytic | 1 | 0 | 1 | 0 | 0.0906 |
| c.510-2A>G |  | splice | I5 | IVS | 0 | 0 | 1 | 0 | 0.0453 |
| c.512G>C | p.G171A | missense | E6 | catalytic | 1 | 0 | 0 | 0 | 0.0453 |
| c.516G>T | p.Q172H | missense | E6 | catalytic | 1 | 0 | 0 | 0 | 0.0453 |
| c.523C>T | p.P175S | missense | E6 | catalytic | 0 | 0 | 1 | 0 | 0.0453 |
| c.526C>T | p.R176* | nonsense | E6 | catalytic | 2 | 4 | 15 | 0 | 0.9511 |
| c.563G>A | p.G188D | missense | E6 | catalytic | 0 | 0 | 1 | 0 | 0.0453 |
| c.598_599insA | p.T200Nfs*6 | insertion | E6 | catalytic | 0 | 0 | 2 | 0 | 0.0906 |
| c.602A>C^#^ | p.H201P | missense | E6 | catalytic | 0 | 0 | 1 | 0 | 0.0453 |
| c.611A>G | p.EX6-96A>G | splice | E6 | catalytic | 24 | 52 | 93 | 4 | 7.8351 |
| c.617A>G | p.Y206C | missense | E6 | catalytic | 0 | 1 | 1 | 0 | 0.0906 |
| c.618C>A | p.Y206* | nonsense | E6 | catalytic | 0 | 0 | 1 | 0 | 0.0453 |
| c.631C>T | p.P211S | missense | E6 | catalytic | 1 | 0 | 0 | 0 | 0.0453 |
| c.641A>G | p.E214G | missense | E6 | catalytic | 1 | 0 | 0 | 0 | 0.0453 |
| c.650G>A | p.C217Y | missense | E6 | catalytic | 0 | 1 | 0 | 0 | 0.0453 |
| c.659A>C | p.H220P | missense | E6 | catalytic | 2 | 0 | 1 | 0 | 0.1359 |
| c.665A>G | p.D222G | missense | E6 | catalytic | 1 | 0 | 0 | 0 | 0.0453 |
| c.668A>T | p.N223I | missense | E6 | catalytic | 1 | 0 | 0 | 0 | 0.0453 |
| c.671T>C | p.I224T | missense | E6 | catalytic | 1 | 0 | 2 | 0 | 0.1359 |
| c.680T>A | p.L227Q | missense | E6 | catalytic | 0 | 0 | 1 | 0 | 0.0453 |
| c.683A>G^#^ | p.E228G | missense | E6 | catalytic | 0 | 1 | 0 | 0 | 0.0453 |
| c.688G>A | p.V230I | missense | E6 | catalytic | 6 | 1 | 0 | 0 | 0.3170 |
| c.691T>C | p.S231P | missense | E6 | catalytic | 0 | 0 | 0 | 1 | 0.0453 |
| c.694C>T | p.Q232* | nonsense | E6 | catalytic | 2 | 3 | 8 | 0 | 0.5888 |
| c.699C>G | p.F233L | missense | E6 | catalytic | 2 | 2 | 0 | 0 | 0.1812 |
| c.707-1G>A |  | splice | I6 | IVS | 0 | 1 | 5 | 0 | 0.2717 |
| c.712A>G | p.T238A | missense | E7 | catalytic | 0 | 0 | 2 | 0 | 0.0906 |
| c.716G>A | p.G239D | missense | E7 | catalytic | 0 | 2 | 1 | 0 | 0.1359 |
| c.716G>C | p.G239A | missense | E7 | catalytic | 0 | 0 | 1 | 0 | 0.0453 |
| c.721C>T | p.R241C | missense | E7 | catalytic | 61 | 62 | 7 | 2 | 5.9783 |
| c.722delG | p.R241Pfs*100 | deletion | E7 | catalytic | 2 | 5 | 9 | 0 | 0.7246 |
| c.722G>A | p.R241H | missense | E7 | catalytic | 4 | 2 | 1 | 0 | 0.3170 |
| c.724C>T | p.L242F | missense | E7 | catalytic | 1 | 0 | 0 | 0 | 0.0453 |
| c.727C>T | p.R243* | nonsense | E7 | catalytic | 0 | 1 | 0 | 0 | 0.0453 |
| c.728G>A | p.R243Q | missense | E7 | catalytic | 59 | 121 | 257 | 9 | 20.1993 |
| c.739G>C | p.G247R | missense | E7 | catalytic | 4 | 11 | 3 | 2 | 0.9058 |
| c.740G>T | p.G247V | missense | E7 | catalytic | 2 | 8 | 12 | 3 | 1.1322 |
| c.754C>T | p.R252W | missense | E7 | catalytic | 0 | 2 | 2 | 0 | 0.1812 |
| c.755G>A | p.R252Q | missense | E7 | catalytic | 2 | 2 | 15 | 1 | 0.9058 |
| c.763T>G | p.L255V | missense | E7 | catalytic | 1 | 0 | 0 | 0 | 0.0453 |
| c.764T>C | p.L255S | missense | E7 | catalytic | 0 | 4 | 6 | 0 | 0.4529 |
| c.770G>T | p.G257V | missense | E7 | catalytic | 0 | 5 | 6 | 0 | 0.4982 |
| c.775G>A | p.A259T | missense | E7 | catalytic | 1 | 0 | 0 | 0 | 0.0453 |
| c.778T>A | p.F260I | missense | E7 | catalytic | 0 | 0 | 1 | 0 | 0.0453 |
| c.781C>T | p.R261* | nonsense | E7 | catalytic | 1 | 1 | 3 | 0 | 0.2264 |
| c.782G>A | p.R261Q | missense | E7 | catalytic | 4 | 12 | 15 | 2 | 1.4946 |
| c.793T>C | p.C265R | missense | E7 | catalytic | 1 | 0 | 0 | 0 | 0.0453 |
| c.795C>A | p.C265* | nonsense | E7 | catalytic | 0 | 0 | 0 | 1 | 0.0453 |
| c.799C>T | p.Q267* | nonsense | E7 | catalytic | 0 | 0 | 1 | 0 | 0.0453 |
| c.800A>T | p.Q267L | missense | E7 | catalytic | 0 | 0 | 0 | 1 | 0.0453 |
| c.809G>A | p.R270K | missense | E7 | catalytic | 0 | 1 | 0 | 0 | 0.0453 |
| c.812A>G | p.H271R | missense | E7 | catalytic | 0 | 0 | 1 | 0 | 0.0453 |
| c.812A>T | p.H271L | missense | E7 | catalytic | 0 | 0 | 1 | 0 | 0.0453 |
| c.824C>T | p.P275L | missense | E7 | catalytic | 0 | 0 | 1 | 0 | 0.0453 |
| c.827T>A | p.M276K | missense | E7 | catalytic | 4 | 2 | 1 | 1 | 0.3623 |
| c.827T>G | p.M276R | missense | E7 | catalytic | 0 | 0 | 1 | 0 | 0.0453 |
| c.832A>G | p.T278A | missense | E7 | catalytic | 2 | 0 | 0 | 0 | 0.0906 |
| c.833C>T | p.T278I | missense | E7 | catalytic | 0 | 1 | 3 | 0 | 0.1812 |
| c.833delC | p.E280Nfs*61 | deletion | E7 | catalytic | 0 | 0 | 1 | 0 | 0.0453 |
| c.838G>A | p.E280K | missense | E7 | catalytic | 0 | 0 | 9 | 0 | 0.4076 |
| c.839A>G | p.E280G | missense | E7 | catalytic | 0 | 0 | 1 | 0 | 0.0453 |
| c.842+1G>A |  | splice | I7 | IVS | 0 | 0 | 1 | 0 | 0.0453 |
| c.842+2T>A |  | splice | I7 | IVS | 0 | 0 | 2 | 0 | 0.0906 |
| c.842C>T | p.P281L | missense | E7 | catalytic | 0 | 2 | 2 | 0 | 0.1812 |
| c.843-14_-11delCTTT |  | deletion | I7 | IVS | 0 | 1 | 3 | 0 | 0.1812 |
| c.845A>G | p.D282G | missense | E8 | catalytic | 1 | 0 | 0 | 0 | 0.0453 |
| c.850T>C | p.C284R | missense | E8 | catalytic | 0 | 0 | 1 | 0 | 0.0453 |
| c.853C>T | p.H285Y | missense | E8 | catalytic | 0 | 0 | 1 | 0 | 0.0453 |
| c.871G>A | p.V291M | missense | E8 | catalytic | 1 | 0 | 0 | 0 | 0.0453 |
| c.874C>T | p.P292S | missense | E8 | catalytic | 0 | 0 | 1 | 0 | 0.0453 |
| c.875C>T | p.P292L | missense | E8 | catalytic | 0 | 0 | 2 | 0 | 0.0906 |
| c.881T>C | p.F294S | missense | E8 | catalytic | 0 | 0 | 1 | 0 | 0.0453 |
| c.886G>C | p.D296H | missense | E8 | catalytic | 1 | 0 | 0 | 0 | 0.0453 |
| c.898G>T | p.A300S | missense | E8 | catalytic | 1 | 0 | 0 | 0 | 0.0453 |
| c.901C>T | p.Q301* | nonsense | E8 | catalytic | 0 | 0 | 1 | 0 | 0.0453 |
| c.907delT | p.S303Pfs*38 | deletion | E8 | catalytic | 0 | 1 | 2 | 0 | 0.1359 |
| c.910C>A | p.Q304K | missense | E8 | catalytic | 0 | 0 | 1 | 0 | 0.0453 |
| c.912+1G>A |  | splice | I8 | IVS | 0 | 1 | 0 | 0 | 0.0453 |
| c.913-2A>G |  | splice | I8 | IVS | 1 | 0 | 1 | 0 | 0.0906 |
| c.913-7A>G |  | splice | I8 | IVS | 0 | 1 | 3 | 0 | 0.1812 |
| c.920G>A | p.G307D | missense | E9 | catalytic | 0 | 1 | 0 | 0 | 0.0453 |
| c.922C>T | p.L308F | missense | E9 | catalytic | 2 | 2 | 0 | 0 | 0.1812 |
| c.929C>T | p.S310F | missense | E9 | catalytic | 0 | 0 | 2 | 0 | 0.0906 |
| c.935G>T | p.G312V | missense | E9 | catalytic | 3 | 0 | 1 | 0 | 0.1812 |
| c.937G>A | p.A313T | missense | E9 | catalytic | 1 | 0 | 0 | 0 | 0.0453 |
| c.940C>G | p.P314A | missense | E9 | catalytic | 1 | 0 | 0 | 0 | 0.0453 |
| c.940C>A | p.P314T | missense | E9 | catalytic | 3 | 0 | 0 | 0 | 0.1359 |
| c.964G>A | p.A322T | missense | E9 | catalytic | 1 | 0 | 0 | 0 | 0.0453 |
| c.965C>A | p.A322D | missense | E9 | catalytic | 1 | 0 | 1 | 0 | 0.0906 |
| c.971T>A | p.I324N | missense | E10 | catalytic | 0 | 2 | 8 | 1 | 0.4982 |
| c.975C>G | p.Y325* | nonsense | E10 | catalytic | 2 | 1 | 7 | 0 | 0.4529 |
| c.977G>A | p.W326* | nonsense | E10 | catalytic | 2 | 1 | 3 | 0 | 0.2717 |
| c.992T>C | p.F331S | missense | E10 | catalytic | 1 | 2 | 0 | 0 | 0.1359 |
| c. 1215_1219delAATAC | p.I406Sfs*17 | deletion | E12 | catalytic | 0 | 1 | 1 | 0 | 0.0906 |
| c.1023delG | p.A342Hfs*59 | deletion | E10 | catalytic | 0 | 0 | 2 | 1 | 0.1359 |
| c.1024G>A | p.A342T | missense | E10 | catalytic | 0 | 1 | 1 | 0 | 0.0906 |
| c.1029T>A | p.Y343* | nonsense | E10 | catalytic | 0 | 1 | 0 | 0 | 0.0453 |
| c.1030G>A | p.G344S | missense | E10 | catalytic | 1 | 0 | 0 | 0 | 0.0453 |
| c.1031G>A | p.G344D | missense | E10 | catalytic | 0 | 0 | 2 | 0 | 0.0906 |
| c.1033G>A | p.A345T | missense | E10 | catalytic | 1 | 2 | 5 | 1 | 0.4076 |
| c.1036G>A | p.G346R | missense | E10 | catalytic | 0 | 0 | 1 | 0 | 0.0453 |
| c.1045T>G | p.S349A | missense | E10 | catalytic | 5 | 8 | 6 | 0 | 0.8605 |
| c.1049C>A | p.S350Y | missense | E10 | catalytic | 0 | 1 | 1 | 0 | 0.0906 |
| c.1065+1G>T |  | splice | I10 | IVS | 0 | 1 | 0 | 0 | 0.0453 |
| c.1066-11G>A |  | splice | I10 | IVS | 1 | 0 | 5 | 0 | 0.2717 |
| c.1066-12delT |  | deletion | I10 | IVS | 0 | 1 | 0 | 0 | 0.0453 |
| c.1066-1G>A |  | splice | I10 | IVS | 0 | 1 | 1 | 0 | 0.0906 |
| c.1066-1G>C |  | splice | I10 | IVS | 0 | 1 | 0 | 0 | 0.0453 |
| c.1066-3C>T |  | splice | I10 | IVS | 0 | 0 | 1 | 0 | 0.0453 |
| c.1068C>A | p.Y356* | nonsense | E11 | catalytic | 16 | 14 | 72 | 5 | 4.8460 |
| c.1071C>A | p.C357* | nonsense | E11 | catalytic | 0 | 0 | 1 | 0 | 0.0453 |
| c.1076C>T | p.S359L | missense | E11 | catalytic | 0 | 0 | 2 | 0 | 0.0906 |
| c.1084C>A | p.P362T | missense | E11 | catalytic | 1 | 1 | 0 | 0 | 0.0906 |
| c.1089delG | p.K363Nfs*37 | deletion | E11 | catalytic | 0 | 1 | 1 | 0 | 0.0906 |
| c.1099C>G | p.L367V | missense | E11 | catalytic | 1 | 0 | 0 | 0 | 0.0453 |
| c.1114A>T | p.T372S | missense | E11 | catalytic | 1 | 0 | 0 | 0 | 0.0453 |
| c.1115C>G | p.T372R | missense | E11 | catalytic | 0 | 0 | 3 | 0 | 0.1359 |
| c.1117G>A | p.A373T | missense | E11 | catalytic | 1 | 0 | 0 | 0 | 0.0453 |
| c.1123C>G | p.Q375E | missense | E11 | catalytic | 2 | 0 | 0 | 0 | 0.0906 |
| c.1125A>C | p.Q375H | missense | E11 | catalytic | 1 | 0 | 0 | 0 | 0.0453 |
| c.1159T>G | p.Y387D | missense | E11 | catalytic | 0 | 1 | 2 | 0 | 0.1359 |
| c.1162G>A | p.V388M | missense | E11 | catalytic | 2 | 4 | 7 | 0 | 0.5888 |
| c.1174T>A | p.F392I | missense | E11 | catalytic | 7 | 0 | 0 | 0 | 0.3170 |
| c.1197A>T | p.V399V | silent | E11 | catalytic | 15 | 32 | 90 | 2 | 6.2953 |
| c.1199+1G>A |  | splice | I11 | IVS | 0 | 0 | 1 | 0 | 0.0453 |
| c.1199+1G>C |  | splice | I11 | IVS | 0 | 0 | 1 | 0 | 0.0453 |
| c.1199G>A | p.R400K | missense | E11 | catalytic | 3 | 3 | 7 | 0 | 0.6341 |
| c.1199G>C | p.R400T | missense | E11 | catalytic | 0 | 1 | 5 | 0 | 0.2717 |
| c.1200-1G>C |  | splice | I11 | IVS | 0 | 0 | 4 | 0 | 0.1812 |
| c.1208C>T | p.A403V | missense | E12 | catalytic | 2 | 0 | 0 | 0 | 0.0906 |
| c.1216A>G | p.I406V | missense | E12 | catalytic | 1 | 0 | 0 | 0 | 0.0453 |
| c.1220C>T | p.P407L | missense | E12 | catalytic | 0 | 1 | 0 | 0 | 0.0453 |
| c.1222C>T | p.R408W | missense | E12 | catalytic | 1 | 2 | 11 | 0 | 0.6341 |
| c.1223G>A | p.R408Q | missense | E12 | catalytic | 13 | 18 | 3 | 2 | 1.6304 |
| c.1238G>C | p.R413P | missense | E12 | oligomerization | 7 | 21 | 83 | 3 | 5.1630 |
| c.1242C>A | p.Y414* | nonsense | E12 | oligomerization | 0 | 0 | 1 | 0 | 0.0453 |
| c.1243G>A | p.D415N | missense | E12 | oligomerization | 3 | 0 | 0 | 0 | 0.1359 |
| c.1243G>T | p.D415Y | missense | E12 | oligomerization | 0 | 0 | 1 | 0 | 0.0453 |
| c.1252A>C | p.T418P | missense | E12 | oligomerization | 5 | 7 | 0 | 0 | 0.5435 |
| c.1256A>G | p.Q419R | missense | E12 | oligomerization | 17 | 0 | 0 | 0 | 0.7699 |
| c.1262T>C | p.I421T | missense | E12 | oligomerization | 3 | 2 | 0 | 0 | 0.2264 |
| c.1289T>C | p.L430P | missense | E12 | oligomerization | 1 | 1 | 1 | 0 | 0.1359 |
| c.1301C>A | p.A434D | missense | E12 | oligomerization | 14 | 23 | 4 | 0 | 1.8569 |
| c.1315+4A>G |  | splice | I12 | IVS | 2 | 1 | 0 | 0 | 0.1359 |
| c.1315+6T>A |  | splice | I12 | IVS | 15 | 4 | 0 | 0 | 0.8605 |
| c.1316-2A>C |  | splice | I12 | IVS | 0 | 4 | 2 | 0 | 0.2717 |
| c.1357-1361delTAAAG | p.*453Pext*33 | deletion | E13 | oligomerization | 1 | 0 | 0 | 0 | 0.0453 |

#: novel mutation

**Table S2. Phenotype–genotype correlation analysis in** **1079 Chinese PKU patients**

| **Classification** | **Genotype** | **PRA%** | **Observed phenotype(No. of patients)** | | | | **Protein domain** |
| --- | --- | --- | --- | --- | --- | --- | --- |
|  |  |  | MHP | mPKU | cPKU | NA |  |
| Homozygotes | p.F39del/p.F39del | null+null | 0 | 0 | 1 | 0 | regulatory+regulatory |
|  | p.H64Dfs*4/p.H64Dfs*4 | null+null | 0 | 0 | 1 | 0 | regulatory+regulatory |
|  | p.S70del/p.S70del | null+null | 0 | 2 | 0 | 0 | regulatory+regulatory |
|  | p.R111*/p.R111* | null+null | 1 | 1 | 4 | 0 | regulatory+regulatory |
|  | C.441+3G>C/C.441+3G>C | null+null | 0 | 1 | 1 | 0 | IVS+IVS |
|  | C.442-1G>A/ C.442-1G>A | null+null | 0 | 0 | 1 | 0 | IVS+IVS |
|  | p.EX6-96A>G/p.EX6-96A>G | null+null | 1 | 2 | 5 | 0 | catalytic+catalytic |
|  | p.Y166*/p.Y166* | null+null | 0 | 0 | 3 | 0 | catalytic+catalytic |
|  | p.Y325*/p.Y325* | null+null | 0 | 0 | 2 | 0 | catalytic+catalytic |
|  | p.Y356*/p.Y356* | null+null | 0 | 0 | 6 | 1 | catalytic+catalytic |
|  | C.1200-1G>C/C.1200-1G>C | null+null | 0 | 0 | 1 | 0 | IVS+IVS |
|  | p.V399V/p.V399V | null+null | 0 | 0 | 4 | 1 | catalytic+catalytic |
|  | p.L255S/p.L255S | 2+2 | 0 | 0 | 1 | 0 | catalytic+catalytic |
|  | p.R252Q/p.R252Q | 3+3 | 0 | 0 | 1 | 0 | catalytic+catalytic |
|  | p.A434D/p.A434D | 3+3 | 0 | 1 | 0 | 0 | oligomerization+oligomerization |
|  | p.F161S/p.F161S | 7+7 | 0 | 0 | 1 | 0 | catalytic+catalytic |
|  | p.R243Q/p.R243Q | 14+14 | 2 | 15 | 41 | 0 | catalytic+catalytic |
|  | p.R241C/p.R241C | 25+25 | 2 | 0 | 0 | 1 | catalytic+catalytic |
|  | p.R413P/p.R413P | 35+35 | 0 | 1 | 4 | 0 | oligomerization+oligomerization |
|  | p.R408Q/p.R408Q | 46+46 | 0 | 1 | 0 | 0 | catalytic+catalytic |
|  | p.R53H/p.R53H | 79+79 | 0 | 0 | 0 | 1 | regulatory+regulatory |
|  | p.T238A/p.T238A | ?+? | 0 | 0 | 1 | 0 | catalytic+catalytic |
|  | p.T278A/p.T278A | ?+? | 1 | 0 | 0 | 0 | catalytic+catalytic |
|  | p.I324N/p.I324N | ?+? | 0 | 0 | 1 | 0 | catalytic+catalytic |
|  | p.S349A/p.S349A | ?+? | 1 | 1 | 0 | 0 | catalytic+catalytic |
|  | p.R400K/p.R400K | ?+? | 0 | 0 | 1 | 0 | catalytic+catalytic |
| Heterozygous | p.S16*/p.V399V | null+null | 1 | 1 | 1 | 0 | regulatory+catalytic |
|  | p.K42del/p.R111* | null+null | 0 | 0 | 1 | 0 | regulatory+regulatory |
|  | C.168+5G>C/p.Y356* | null+null | 0 | 0 | 1 | 0 | IVS+catalytic |
|  | C.168+5G>C/p.V399V | null+null | 0 | 1 | 0 | 0 | IVS+catalytic |
|  | p.L62Pfs*7/p.Y356* | null+null | 0 | 0 | 1 | 0 | regulatory+catalytic |
|  | p.S70del/C.1066-1G>A | null+null | 0 | 0 | 1 | 0 | regulatory+IVS |
|  | p.S70del/p.V399V | null+null | 0 | 3 | 1 | 0 | regulatory+catalytic |
|  | p.S70del/p.R111* | null+null | 0 | 2 | 3 | 0 | regulatory+regulatory |
|  | p.S70del/p.R176* | null+null | 0 | 1 | 0 | 0 | regulatory+catalytic |
|  | p.S70del/p.EX6-96A>G | null+null | 0 | 3 | 0 | 0 | regulatory+catalytic |
|  | p.S70del/p.Y356* | null+null | 0 | 1 | 1 | 0 | regulatory+catalytic |
|  | p.S70del/C.1315+6T>A | null+null | 1 | 0 | 0 | 0 | regulatory+IVS |
|  | p.I95del/p.R111* | null+null | 0 | 1 | 0 | 0 | regulatory+regulatory |
|  | p.R111*/p.Y356* | null+null | 0 | 0 | 2 | 2 | regulatory+catalytic |
|  | p.R111*/p.V399V | null+null | 0 | 1 | 7 | 0 | regulatory+catalytic |
|  | p.R111*/C.1199+1G>C | null+null | 0 | 0 | 1 | 0 | regulatory+IVS |
|  | p.R111*/IVS3-2A>T | null+null | 0 | 0 | 1 | 0 | regulatory+IVS |
|  | p.R111*/C.442-1G>A | null+null | 0 | 0 | 3 | 0 | regulatory+IVS |
|  | p.R111*/p.Y166* | null+null | 0 | 0 | 1 | 0 | regulatory+catalytic |
|  | p.R111*/p.EX6-96A>G | null+null | 2 | 1 | 9 | 0 | regulatory+catalytic |
|  | p.R111*/C.842+1G>A | null+null | 0 | 0 | 1 | 0 | regulatory+IVS |
|  | p.R111*/p.W326* | null+null | 0 | 0 | 1 | 0 | regulatory+catalytic |
|  | p.R111*/C.1315+6T>A | null+null | 1 | 0 | 0 | 0 | regulatory+IVS |
|  | C.441+1G>A/p.EX6-96A>G | null+null | 0 | 0 | 1 | 0 | IVS+catalytic |
|  | C.441+2T>A/p.V399V | null+null | 1 | 0 | 0 | 0 | IVS+catalytic |
|  | C.441+2T>A/p.Y166* | null+null | 0 | 0 | 1 | 0 | IVS+catalytic |
|  | C.442-1G>A/C.441+3G>C | null+null | 0 | 0 | 2 | 0 | IVS+IVS |
|  | C.441+3G>C/p.EX6-96A>G | null+null | 0 | 0 | 1 | 0 | IVS+catalytic |
|  | C.442-1G>A/p.R176* | null+null | 0 | 0 | 2 | 0 | IVS+catalytic |
|  | C.442-1G>A/C.1066-1G>A | null+null | 0 | 1 | 0 | 0 | IVS+IVS |
|  | C.442-1G>A/p.Y356* | null+null | 1 | 1 | 5 | 0 | IVS+catalytic |
|  | C.442-1G>A/p.V399V | null+null | 0 | 0 | 6 | 0 | IVS+catalytic |
|  | p.S70del/C.442-1G>A | null+null | 0 | 0 | 1 | 0 | regulatory+IVS |
|  | C.442-1G>A/p.EX6-96A>G | null+null | 0 | 0 | 2 | 0 | IVS+catalytic |
|  | C.442-1G>A/p.Q232* | null+null | 0 | 0 | 2 | 0 | IVS+catalytic |
|  | C.442-1G>A/C.707-1G>A | null+null | 0 | 0 | 1 | 0 | IVS+IVS |
|  | C.442-1G>A/p.R241Pfs*100 | null+null | 0 | 0 | 1 | 0 | IVS+catalytic |
|  | C.442-1G>A/p.Q267* | null+null | 0 | 0 | 1 | 0 | IVS+catalytic |
|  | C.442-1G>A/p.W326* | null+null | 0 | 0 | 1 | 0 | IVS+catalytic |
|  | C.442-1G>A/C.1315+6T>A | null+null | 1 | 0 | 0 | 0 | IVS+IVS |
|  | p.Y166*/p.EX6-96A>G | null+null | 0 | 0 | 3 | 0 | catalytic+catalytic |
|  | p.Y166*/p.R241Pfs*100 | null+null | 0 | 0 | 1 | 0 | catalytic+catalytic |
|  | p.Y166*/p.R176* | null+null | 0 | 0 | 1 | 0 | catalytic+catalytic |
|  | p.Y166*/C.1315+6T>A | null+null | 1 | 0 | 0 | 0 | catalytic+IVS |
|  | p.Y166*/p.V399V | null+null | 0 | 1 | 0 | 0 | catalytic+catalytic |
|  | p.R176*/p.Y356* | null+null | 0 | 0 | 3 | 0 | catalytic+catalytic |
|  | p.R176*/C.1315+6T>A | null+null | 0 | 1 | 0 | 0 | catalytic+IVS |
|  | p.R176*/p.EX6-96A>G | null+null | 0 | 0 | 2 | 0 | catalytic+catalytic |
|  | p.R176*/C.707-1G>A | null+null | 0 | 0 | 1 | 0 | catalytic+IVS |
|  | p.EX6-96A>G/C.1066-11G>A | null+null | 0 | 0 | 1 | 0 | catalytic+IVS |
|  | p.EX6-96A>G/p.Y356* | null+null | 1 | 0 | 5 | 0 | catalytic+catalytic |
|  | p.EX6-96A>G/p.K363Nfs*37 | null+null | 0 | 0 | 1 | 0 | catalytic+catalytic |
|  | p.EX6-96A>G/p.V399V | null+null | 0 | 0 | 11 | 0 | catalytic+catalytic |
|  | p.EX6-96A>G/C.1199+1G>A | null+null | 0 | 0 | 1 | 0 | catalytic+IVS |
|  | p.EX6-96A>G/C.1315+6T>A | null+null | 3 | 1 | 0 | 0 | catalytic+IVS |
|  | p.EX6-96A>G/C.707-1G>A | null+null | 0 | 0 | 1 | 0 | catalytic+IVS |
|  | p.EX6-96A>G/p.Q232* | null+null | 0 | 0 | 1 | 0 | catalytic+catalytic |
|  | p.EX6-96A>G/p.R241Pfs*100 | null+null | 0 | 1 | 1 | 0 | catalytic+catalytic |
|  | p.EX6-96A>G/C.912+1G>A | null+null | 0 | 1 | 0 | 0 | catalytic+IVS |
|  | p.EX6-96A>G/C.913-7A>G | null+null | 0 | 1 | 0 | 0 | catalytic+IVS |
|  | p.EX6-96A>G/p.Y325* | null+null | 0 | 0 | 1 | 0 | catalytic+catalytic |
|  | p.EX6-96A>G/p.W326* | null+null | 0 | 0 | 1 | 0 | catalytic+catalytic |
|  | p.Y206*/p.V399V | null+null | 0 | 0 | 1 | 0 | catalytic+catalytic |
|  | p.Q232*/p.A342Hfs*59 | null+null | 0 | 0 | 1 | 0 | catalytic+catalytic |
|  | p.R252W/p.V399V | null+null | 0 | 1 | 1 | 0 | catalytic+catalytic |
|  | p.R252W/C.707-1G>A | null+null | 0 | 1 | 0 | 0 | catalytic+IVS |
|  | p.R261*/p.V399V | null+null | 1 | 1 | 0 | 0 | catalytic+catalytic |
|  | p.R261*/C.1200-1G>C | null+null | 0 | 0 | 1 | 0 | catalytic+IVS |
|  | C.843-14_-11delCTTT/p.Y356* | null+null | 0 | 1 | 0 | 0 | IVS+catalytic |
|  | c.843-14_-11delCTTT /p.Y325* | null+null | 0 | 0 | 1 | 0 | IVS+catalytic |
|  | C.842+2T>A/C.1066-11G>A | null+null | 0 | 0 | 1 | 0 | IVS+IVS |
|  | p.S303Pfs*38/p.V399V | null+null | 0 | 1 | 0 | 0 | catalytic+catalytic |
|  | C.913-7A>G/p.Y356* | null+null | 0 | 0 | 1 | 0 | IVS+catalytic |
|  | C.913-7A>G/p.V399V | null+null | 0 | 0 | 1 | 0 | IVS+catalytic |
|  | C.1066-12delT/p.Y356* | null+null | 0 | 1 | 0 | 0 | IVS+catalytic |
|  | C.1066-3C>T/p.V399V | null+null | 0 | 0 | 1 | 0 | IVS+catalytic |
|  | p.Y356*/p.C357* | null+null | 0 | 0 | 1 | 0 | catalytic+catalytic |
|  | p.Y356*/p.V399V | null+null | 0 | 1 | 7 | 0 | catalytic+catalytic |
|  | p.Y356*/C.1315+4A>G | null+null | 0 | 1 | 0 | 0 | catalytic+IVS |
|  | p.Y356*/C.1316-2A>C | null+null | 0 | 0 | 1 | 0 | catalytic+IVS |
|  | p.Y356*/p.*453Pext*33 | null+null | 1 | 0 | 0 | 0 | catalytic+oligomerization |
|  | p.V399V/p.E43* | null+null | 0 | 0 | 1 | 0 | catalytic+regulatory |
|  | p.V399V/C.1315+6T>A | null+null | 2 | 0 | 0 | 0 | catalytic+IVS |
|  | p.G257V/p.V399V | 1+null | 0 | 0 | 2 | 0 | catalytic+catalytic |
|  | p.G257V/p.S70del/ | 1+null | 0 | 1 | 1 | 0 | catalytic+regulatory |
|  | p.G257V/C.442-1G>A | 1+null | 0 | 0 | 1 | 0 | catalytic+IVS |
|  | p.R158W/C.1065+1G>T | 2+null | 0 | 1 | 0 | 0 | catalytic+IVS |
|  | p.R158W/p.EX6-96A>G | 2+null | 0 | 1 | 1 | 0 | catalytic+catalytic |
|  | p.R158W/p.E280Nfs*61 | 2+null | 0 | 0 | 1 | 0 | catalytic+catalytic |
|  | p.L255S/p.Y166* | 2+null | 0 | 1 | 0 | 0 | catalytic+catalytic |
|  | p.L255S/p.R176* | 2+null | 0 | 0 | 1 | 0 | catalytic+catalytic |
|  | p.L255S/p.EX6-96A>G | 2+null | 0 | 0 | 1 | 0 | catalytic+catalytic |
|  | p.L255S/p.W326* | 2+null | 0 | 1 | 0 | 0 | catalytic+catalytic |
|  | p.L255S/p.V399V | 2+null | 0 | 0 | 1 | 0 | catalytic+catalytic |
|  | p.E280K/p.R111* | 2+null | 0 | 0 | 1 | 0 | catalytic+regulatory |
|  | p.E280K/C.442-1G>A | 2+null | 0 | 0 | 1 | 0 | regulatory+IVS |
|  | p.E280K/p.Y166* | 2+null | 0 | 0 | 1 | 0 | catalytic+catalytic |
|  | p.E280K/p.EX6-96A>G | 2+null | 0 | 0 | 1 | 0 | catalytic+catalytic |
|  | p.E280K/p.S303Pfs*38 | 2+null | 0 | 0 | 1 | 0 | catalytic+catalytic |
|  | p.E280K/p.V399V | 2+null | 0 | 0 | 1 | 0 | catalytic+catalytic |
|  | p.P281L/p.EX6-96A>G | 2+null | 0 | 0 | 1 | 0 | catalytic+catalytic |
|  | p.R408W/p.Y166* | 2+null | 0 | 0 | 1 | 0 | catalytic+catalytic |
|  | p.R408W/p.EX6-96A>G | 2+null | 1 | 0 | 0 | 0 | catalytic+catalytic |
|  | p.R252Q/p.S70del | 3+null | 0 | 0 | 1 | 0 | catalytic+regulatory |
|  | p.R252Q/p.R111* | 3+null | 0 | 0 | 1 | 0 | catalytic+regulatory |
|  | p.R252Q/p.EX6-96A>G | 3+null | 0 | 0 | 2 | 0 | catalytic+catalytic |
|  | p.R252Q/p.S303Pfs*38 | 3+null | 0 | 0 | 1 | 0 | catalytic+catalytic |
|  | p.R252Q/p.V399V | 3+null | 0 | 0 | 3 | 0 | catalytic+catalytic |
|  | p.A434D/p.R111* | 3+null | 2 | 1 | 0 | 0 | oligomerization+regulatory |
|  | p.A434D/C.442-1G>A | 3+null | 2 | 2 | 0 | 0 | oligomerization+IVS |
|  | p.A434D/p.R176* | 3+null | 1 | 0 | 0 | 0 | oligomerization+catalytic |
|  | p.A434D/p.EX6-96A>G | 3+null | 1 | 4 | 0 | 0 | oligomerization+catalytic |
|  | p.A434D/p.Y325* | 3+null | 0 | 1 | 0 | 0 | oligomerization+catalytic |
|  | p.A434D/p.Y356* | 3+null | 1 | 0 | 0 | 0 | oligomerization+catalytic |
|  | p.A434D/p.V399V | 3+null | 0 | 1 | 1 | 0 | oligomerization+catalytic |
|  | p.G247V/ c.169+19T>C | 4+null | 0 | 1 | 0 | 0 | catalytic+IVS |
|  | p.G247V/p.R111* | 4+null | 0 | 0 | 0 | 1 | catalytic+regulatory |
|  | p.G247V/C.442-1G>A | 4+null | 0 | 0 | 1 | 0 | catalytic+IVS |
|  | p.G247V/p.EX6-96A>G | 4+null | 0 | 1 | 1 | 0 | catalytic+catalytic |
|  | p.G247V/p.A342Hfs*59 | 4+null | 0 | 0 | 0 | 1 | catalytic+catalytic |
|  | p.G247V/p.Y356* | 4+null | 0 | 0 | 1 | 0 | catalytic+catalytic |
|  | p.G247V/p.V399V | 4+null | 0 | 0 | 3 | 0 | catalytic+catalytic |
|  | p.F161S/p.R111* | 7+null | 0 | 0 | 1 | 0 | catalytic+regulatory |
|  | p.F161S/C.442-1G>A | 7+null | 0 | 0 | 2 | 0 | catalytic+IVS |
|  | p.F161S/p.EX6-96A>G | 7+null | 0 | 0 | 2 | 0 | catalytic+catalytic |
|  | p.F161S/p.R241Pfs*100 | 7+null | 0 | 0 | 1 | 0 | catalytic+catalytic |
|  | p.F161S/p.Y356* | 7+null | 0 | 0 | 2 | 0 | catalytic+catalytic |
|  | p.F161S/p.V399V | 7+null | 0 | 1 | 0 | 0 | catalytic+catalytic |
|  | p.R158Q/p.R111* | 10+null | 0 | 0 | 1 | 0 | catalytic+regulatory |
|  | p.R158Q/p.EX6-96A>G | 10+null | 0 | 1 | 1 | 0 | catalytic+catalytic |
|  | p.R158Q/p.Y356* | 10+null | 0 | 0 | 1 | 0 | catalytic+catalytic |
|  | p.R270K/p.EX6-96A>G | 11+null | 0 | 1 | 0 | 0 | catalytic+catalytic |
|  | p.R243Q/p.R13Qfs*5 | 14+null | 0 | 0 | 1 | 0 | catalytic+regulatory |
|  | p.R243Q/p.S16* | 14+null | 0 | 1 | 0 | 0 | catalytic+regulatory |
|  | p.R243Q/c.60+5_+6delGC | 14+null | 0 | 0 | 1 | 0 | catalytic+IVS |
|  | p.R243Q/p.F39del | 14+null | 0 | 0 | 3 | 0 | catalytic+regulatory |
|  | p.R243Q/p.E43* | 14+null | 0 | 0 | 1 | 0 | catalytic+regulatory |
|  | p.R243Q/p.S70del | 14+null | 1 | 7 | 1 | 0 | catalytic+regulatory |
|  | p.R243Q/p.I95del | 14+null | 0 | 1 | 1 | 0 | catalytic+regulatory |
|  | p.R243Q/p.R111* | 14+null | 1 | 0 | 18 | 1 | catalytic+regulatory |
|  | p.R243Q/IVS3-2A>T | 14+null | 0 | 0 | 1 | 0 | catalytic+IVS |
|  | p.R243Q/C.441+3G>C | 14+null | 0 | 1 | 1 | 0 | catalytic+IVS |
|  | p.R243Q/C.442-1G>A | 14+null | 1 | 2 | 16 | 0 | catalytic+IVS |
|  | p.R243Q/p.Y166* | 14+null | 0 | 0 | 5 | 0 | catalytic+catalytic |
|  | p.R243Q/p.R176* | 14+null | 0 | 0 | 3 | 0 | catalytic+catalytic |
|  | p.R243Q/p.T200Nfs*6 | 14+null | 0 | 0 | 1 | 0 | catalytic+catalytic |
|  | p.R243Q/p.EX6-96A>G | 14+null | 1 | 7 | 15 | 2 | catalytic+catalytic |
|  | p.R243Q/p.Q232* | 14+null | 0 | 1 | 1 | 0 | catalytic+catalytic |
|  | p.R243Q/C.707-1G>A | 14+null | 0 | 0 | 2 | 0 | catalytic+IVS |
|  | p.R243Q/p.R241Pfs*100 | 14+null | 0 | 2 | 2 | 0 | catalytic+catalytic |
|  | p.R243Q/p.R261* | 14+null | 0 | 0 | 1 | 0 | catalytic+catalytic |
|  | p.R243Q/c.843-14_-11delCTTT | 14+null | 0 | 0 | 1 | 0 | catalytic+IVS |
|  | p.R243Q/C.913-2A>G | 14+null | 0 | 0 | 1 | 0 | catalytic+IVS |
|  | p.R243Q/C.1066-11G>A | 14+null | 0 | 0 | 1 | 0 | catalytic+IVS |
|  | p.R243Q/p.Y356* | 14+null | 0 | 4 | 9 | 1 | catalytic+catalytic |
|  | p.R243Q/p.K363Nfs*37 | 14+null | 0 | 1 | 0 | 0 | catalytic+catalytic |
|  | p.R243Q/p.V399V | 14+null | 1 | 9 | 18 | 0 | catalytic+catalytic |
|  | p.R243Q/C.1200-1G>C | 14+null | 0 | 0 | 1 | 0 | catalytic+IVS |
|  | p.R243Q/C.1315+4A>G | 14+null | 1 | 0 | 0 | 0 | catalytic+IVS |
|  | p.R243Q/C.1315+6T>A | 14+null | 3 | 1 | 0 | 0 | catalytic+IVS |
|  | p.R243Q/C.1316-2A>C | 14+null | 0 | 0 | 1 | 0 | catalytic+IVS |
|  | p.R241H/p.V399V | 23+null | 1 | 0 | 0 | 0 | catalytic+catalytic |
|  | p.R241H/p.EX6-96A>G | 23+null | 0 | 1 | 0 | 0 | catalytic+catalytic |
|  | p.R241C/p.S70del | 25+null | 1 | 0 | 0 | 0 | catalytic+regulatory |
|  | p.R241C/p.R111* | 25+null | 3 | 7 | 0 | 0 | catalytic+regulatory |
|  | p.R241C/ c.353-2A>G | 25+null | 1 | 0 | 0 | 0 | catalytic+IVS |
|  | p.R241C/C.441+3G>C | 25+null | 0 | 1 | 0 | 0 | catalytic+IVS |
|  | p.R241C/C.442-1G>A | 25+null | 0 | 6 | 0 | 0 | catalytic+IVS |
|  | p.R241C/p.Q160* | 25+null | 1 | 0 | 0 | 0 | catalytic+catalytic |
|  | p.R241C/p.Y166* | 25+null | 0 | 1 | 0 | 0 | catalytic+catalytic |
|  | p.R241C/p.R176* | 25+null | 1 | 1 | 0 | 0 | catalytic+catalytic |
|  | p.R241C/p.EX6-96A>G | 25+null | 5 | 9 | 2 | 0 | catalytic+catalytic |
|  | p.R241C/p.Q232* | 25+null | 1 | 0 | 0 | 0 | catalytic+catalytic |
|  | p.R241C/p.R241Pfs*100 | 25+null | 0 | 1 | 0 | 0 | catalytic+catalytic |
|  | p.R241C/C.913-2A>G | 25+null | 1 | 0 | 0 | 0 | catalytic+IVS |
|  | p.R241C/p.Y325* | 25+null | 1 | 0 | 0 | 0 | catalytic+catalytic |
|  | p.R241C/p.Y343* | 25+null | 0 | 1 | 0 | 0 | catalytic+catalytic |
|  | p.R241C/C.1066-1G>C | 25+null | 0 | 1 | 0 | 0 | catalytic+IVS |
|  | p.R241C/p.Y356* | 25+null | 4 | 1 | 1 | 0 | catalytic+catalytic |
|  | p.R241C/p.V399V | 25+null | 2 | 5 | 1 | 0 | catalytic+catalytic |
|  | p.R241C/C.1316-2A>C | 25+null | 0 | 1 | 0 | 0 | catalytic+IVS |
|  | p.A342T/C.442-1G>A | 26+null | 0 | 1 | 0 | 0 | catalytic+IVS |
|  | p.A342T/p.Y356* | 26+null | 0 | 0 | 1 | 0 | catalytic+catalytic |
|  | p.I95del/C.1316-2A>C | 27+null | 0 | 1 | 0 | 0 | regulatory+IVS |
|  | p.I95del/p.S70del | 27+null | 0 | 1 | 0 | 0 | regulatory+regulatory |
|  | p.G171A/p.Y356* | 27+null | 1 | 0 | 0 | 0 | catalytic+catalytic |
|  | p.V388M/p.Y166* | 28+null | 0 | 0 | 1 | 0 | catalytic+catalytic |
|  | p.V388M/C.442-1G>A | 28+null | 0 | 0 | 1 | 0 | catalytic+IVS |
|  | p.V388M/p.R241Pfs*100 | 28+null | 0 | 1 | 1 | 0 | catalytic+catalytic |
|  | p.V388M/p.Y356* | 28+null | 0 | 0 | 1 | 0 | catalytic+catalytic |
|  | p.A300S/p.Y356* | 31+null | 1 | 0 | 0 | 0 | catalytic+catalytic |
|  | p.I65T/p.R111* | 33+null | 0 | 1 | 2 | 0 | regulatory+regulatory |
|  | p.I65T/p.EX6-96A>G | 33+null | 0 | 0 | 1 | 0 | regulatory+catalytic |
|  | p.I65T/p.Y356* | 33+null | 0 | 0 | 2 | 0 | regulatory+catalytic |
|  | p.I65T/p.I406Sfs*17 | 33+null | 0 | 0 | 1 | 0 | regulatory+catalytic |
|  | p.R413P/p.S16* | 35+null | 0 | 0 | 1 | 0 | oligomerization+regulatory |
|  | p.R413P/p.S70del | 35+null | 0 | 3 | 1 | 0 | oligomerization+regulatory |
|  | p.R413P/p.I95del | 35+null | 0 | 0 | 1 | 0 | oligomerization+regulatory |
|  | p.R413P/p.R111* | 35+null | 0 | 2 | 4 | 0 | oligomerization+regulatory |
|  | p.R413P/C.442-1G>A | 35+null | 0 | 0 | 4 | 0 | oligomerization+IVS |
|  | p.R413P/p.Y166* | 35+null | 0 | 0 | 1 | 1 | oligomerization+catalytic |
|  | p.R413P/p.EX6-96A>G | 35+null | 0 | 1 | 6 | 1 | oligomerization+catalytic |
|  | p.R413P/p.Q232* | 35+null | 0 | 1 | 1 | 0 | oligomerization+catalytic |
|  | p.R413P/c.843-14_-11delCTTT | 35+null | 0 | 0 | 1 | 0 | oligomerization+IVS |
|  | p.R413P/p.Y356* | 35+null | 0 | 0 | 7 | 0 | oligomerization+catalytic |
|  | p.R413P/p.V399V | 35+null | 0 | 1 | 7 | 0 | oligomerization+catalytic |
|  | p.R413P/C.1315+6T>A | 35+null | 1 | 1 | 0 | 0 | oligomerization+IVS |
|  | p.R261Q/p.R111* | 44+null | 0 | 1 | 0 | 0 | catalytic+regulatory |
|  | p.R261Q/C.442-1G>A | 44+null | 0 | 0 | 1 | 0 | catalytic+IVS |
|  | p.R261Q/p.Y166* | 44+null | 0 | 0 | 1 | 0 | catalytic+catalytic |
|  | p.R261Q/C.510-2A>G | 44+null | 0 | 0 | 1 | 0 | catalytic+IVS |
|  | p.R261Q/p.EX6-96A>G | 44+null | 0 | 2 | 1 | 0 | catalytic+catalytic |
|  | p.R261Q/p.C265* | 44+null | 0 | 0 | 0 | 1 | catalytic+catalytic |
|  | p.R261Q/p.Q301* | 44+null | 0 | 0 | 1 | 0 | catalytic+catalytic |
|  | p.R261Q/p.Y356* | 44+null | 0 | 1 | 1 | 0 | catalytic+catalytic |
|  | p.R261Q/p.V399V | 44+null | 0 | 1 | 2 | 0 | catalytic+catalytic |
|  | p.R261Q/C.1315+4A>G | 44+null | 1 | 0 | 0 | 0 | catalytic+IVS |
|  | p.R408Q/C.441+3G>C | 46+null | 0 | 1 | 0 | 0 | catalytic+IVS |
|  | p.R408Q/C.442-1G>A | 46+null | 0 | 2 | 0 | 0 | catalytic+IVS |
|  | p.R408Q/p.R241Pfs*100 | 46+null | 0 | 0 | 1 | 0 | catalytic+catalytic |
|  | p.R408Q/p.R243* | 46+null | 0 | 1 | 0 | 0 | catalytic+catalytic |
|  | p.R408Q/p.Y356* | 46+null | 1 | 0 | 0 | 0 | catalytic+catalytic |
|  | p.R408Q/C.1315+6T>A | 46+null | 1 | 0 | 0 | 0 | catalytic+IVS |
|  | p.R408Q/C.1316-2A>C | 46+null | 0 | 1 | 0 | 0 | catalytic+IVS |
|  | p.L308F/p.S70del | 49+null | 1 | 0 | 0 | 0 | catalytic+regulatory |
|  | p.L308F/C.442-1G>A | 49+null | 0 | 1 | 0 | 0 | catalytic+IVS |
|  | p.L308F/p.Q232* | 49+null | 0 | 1 | 0 | 0 | catalytic+catalytic |
|  | p.Q419R/p.R111* | 71+null | 1 | 0 | 0 | 0 | oligomerization+regulatory |
|  | p.Q419R/p.EX6-96A>G | 71+null | 3 | 0 | 0 | 0 | oligomerization+catalytic |
|  | p.Q419R/p.Q232* | 71+null | 1 | 0 | 0 | 0 | oligomerization+catalytic |
|  | p.Q419R/p.W326* | 71+null | 1 | 0 | 0 | 0 | oligomerization+catalytic |
|  | p.Q419R/p.Y356* | 71+null | 3 | 0 | 0 | 0 | oligomerization+catalytic |
|  | p.Q419R/p.V399V | 71+null | 1 | 0 | 0 | 0 | oligomerization+catalytic |
|  | p.D415N/p.Y356* | 72+null | 1 | 0 | 0 | 0 | catalytic+oligomerization |
|  | p.A313T/C.442-1G>A | 76+null | 1 | 0 | 0 | 0 | catalytic+IVS |
|  | p.R53H/p.S70del | 79+null | 0 | 1 | 0 | 0 | regulatory+regulatory |
|  | p.R53H/p.R111* | 79+null | 0 | 1 | 0 | 0 | regulatory+regulatory |
|  | p.R53H/C.442-1G>A | 79+null | 1 | 0 | 0 | 0 | regulatory+IVS |
|  | p.R53H/C.842+2T>A | 79+null | 0 | 0 | 1 | 0 | regulatory+IVS |
|  | p.R53H/C.1066-11G>A | 79+null | 0 | 0 | 1 | 0 | regulatory+IVS |
|  | p.R53H/p.Y356* | 79+null | 1 | 0 | 0 | 0 | regulatory+catalytic |
|  | p.R53H/p.V399V | 79+null | 0 | 0 | 1 | 0 | regulatory+catalytic |
|  | p.M1I/p.EX6-96A>G | ?+null | 0 | 1 | 0 | 0 | regulatory+catalytic |
|  | p.A47E/p.EX6-96A>G | ?+null | 0 | 1 | 0 | 0 | regulatory+catalytic |
|  | p.E56D/p.V399V | ?+null | 0 | 0 | 1 | 0 | regulatory+catalytic |
|  | p.E56D/C.442-1G>A | ?+null | 0 | 0 | 1 | 0 | regulatory+IVS |
|  | p.I65V/p.EX6-96A>G | ?+null | 1 | 0 | 0 | 0 | regulatory+catalytic |
|  | p.L98V/p.Y356* | ?+null | 0 | 0 | 1 | 0 | regulatory+catalytic |
|  | p.D101N/p.R111* | ?+null | 1 | 0 | 0 | 0 | regulatory+regulatory |
|  | p.H107R/p.R241Pfs*100 | ?+null | 1 | 0 | 0 | 0 | regulatory+catalytic |
|  | p.H107R/p.S70del | ?+null | 1 | 0 | 0 | 0 | regulatory+regulatory |
|  | p.F121V/p.R111*/ | ?+null | 0 | 1 | 0 | 0 | regulatory+regulatory |
|  | p.P147L/p.I406Sfs*17 | ?+null | 0 | 1 | 0 | 0 | catalytic+catalytic |
|  | p.P147L/p.EX6-96A>G | ?+null | 0 | 2 | 0 | 0 | catalytic+catalytic |
|  | p.Y154H/p.R111* | ?+null | 0 | 0 | 1 | 0 | regulatory+catalytic |
|  | p.A156P/p.V5Sfs*33 | ?+null | 0 | 0 | 1 | 0 | catalytic+regulatory |
|  | p.A165D/C.1066-11G>A | ?+null | 0 | 0 | 1 | 0 | catalytic+IVS |
|  | p.R169G/p.R241Pfs*100 | ?+null | 1 | 0 | 0 | 0 | catalytic+catalytic |
|  | p.Y206C/p.Y325* | ?+null | 0 | 0 | 1 | 0 | catalytic+catalytic |
|  | p.C217Y/p.R111* | ?+null | 0 | 1 | 0 | 0 | catalytic+regulatory |
|  | p.D222G/p.S16* | ?+null | 1 | 0 | 0 | 0 | catalytic+regulatory |
|  | p.N223I/p.Y166* | ?+null | 1 | 0 | 0 | 0 | catalytic+catalytic |
|  | p.I224T/p.Q232* | ?+null | 0 | 0 | 1 | 0 | catalytic+catalytic |
|  | p.L227Q/p.EX6-96A>G | ?+null | 0 | 0 | 1 | 0 | catalytic+catalytic |
|  | p.E228G/p.EX6-96A>G | ?+null | 0 | 1 | 0 | 0 | catalytic+catalytic |
|  | p.F233L/C.442-1G>A | ?+null | 1 | 0 | 0 | 0 | catalytic+IVS |
|  | p.G239D/p.R241Pfs*100 | ?+null | 0 | 0 | 1 | 0 | catalytic+catalytic |
|  | p.H201P/p.Y356* | ?+null | 0 | 0 | 1 | 0 | catalytic+catalytic |
|  | p.G247R/p.S16* | ?+null | 0 | 0 | 1 | 0 | catalytic+regulatory |
|  | p.G247R/p.EX6-96A>G | ?+null | 1 | 1 | 0 | 0 | catalytic+catalytic |
|  | p.G247R/C.442-1G>A | ?+null | 0 | 1 | 0 | 0 | catalytic+IVS |
|  | p.G247R/p.R111* | ?+null | 1 | 1 | 0 | 0 | catalytic+regulatory |
|  | p.G247R/p.Y356* | ?+null | 0 | 1 | 0 | 0 | catalytic+catalytic |
|  | p.G247R/C.1316-2A>C | ?+null | 0 | 1 | 0 | 0 | catalytic+IVS |
|  | p.H271L/C.168+5G>C | ?+null | 0 | 0 | 1 | 0 | catalytic+IVS |
|  | p.M276K/p.Y166* | ?+null | 0 | 0 | 1 | 0 | catalytic+catalytic |
|  | p.M276K/p.EX6-96A>G | ?+null | 1 | 1 | 0 | 0 | catalytic+catalytic |
|  | p.M276K/p.V399V | ?+null | 1 | 0 | 0 | 0 | catalytic+catalytic |
|  | p.M276R/C.442-1G>A | ?+null | 0 | 0 | 1 | 0 | catalytic+IVS |
|  | p.E280G/p.V399V | ?+null | 0 | 0 | 1 | 0 | catalytic+catalytic |
|  | p.D282G/p.V399V | ?+null | 1 | 0 | 0 | 0 | catalytic+catalytic |
|  | p.C284R/p.EX6-96A>G | ?+null | 0 | 0 | 1 | 0 | catalytic+catalytic |
|  | p.H285Y/p.A342Hfs*59 | ?+null | 0 | 0 | 1 | 0 | catalytic+catalytic |
|  | p.V291M/p.R111* | ?+null | 1 | 0 | 0 | 0 | catalytic+regulatory |
|  | p.P292L/C.913-7A>G | ?+null | 0 | 0 | 1 | 0 | catalytic+IVS |
|  | p.F294S/p.R111* | ?+null | 0 | 0 | 1 | 0 | catalytic+regulatory |
|  | p.Q304K/p.R261* | ?+null | 0 | 0 | 1 | 0 | catalytic+catalytic |
|  | p.G307D/p.R111* | ?+null | 0 | 1 | 0 | 0 | catalytic+regulatory |
|  | p.S310F/p.R111* | ?+null | 0 | 0 | 1 | 0 | catalytic+regulatory |
|  | p.S310F/p.Y356* | ?+null | 0 | 0 | 1 | 0 | catalytic+catalytic |
|  | p.P314T/p.V399V | ?+null | 1 | 0 | 0 | 0 | catalytic+catalytic |
|  | p.P314A/p.Y356* | ?+null | 1 | 0 | 0 | 0 | catalytic+catalytic |
|  | p.A322T/p.R111* | ?+null | 1 | 0 | 0 | 0 | catalytic+regulatory |
|  | p.I324N/p.R111* | ?+null | 0 | 0 | 1 | 0 | catalytic+regulatory |
|  | p.I324N/c.509+1G>A | ?+null | 0 | 0 | 1 | 0 | catalytic+IVS |
|  | p.I324N/p.EX6-96A>G | ?+null | 0 | 0 | 0 | 1 | catalytic+catalytic |
|  | p.I324N/p.Y356* | ?+null | 0 | 0 | 1 | 0 | catalytic+catalytic |
|  | p.I324N/p.V399V | ?+null | 0 | 1 | 0 | 0 | catalytic+catalytic |
|  | p.F331S/p.S16* | ?+null | 0 | 1 | 0 | 0 | catalytic+regulatory |
|  | p.F331S/C.442-1G>A | ?+null | 0 | 1 | 0 | 0 | catalytic+IVS |
|  | p.A322D/C.442-1G>A | ?+null | 0 | 0 | 1 | 0 | catalytic+IVS |
|  | p.G344D/p.Q232* | ?+null | 0 | 0 | 1 | 0 | catalytic+catalytic |
|  | p.A345T/p.EX6-96A>G | ?+null | 0 | 0 | 1 | 0 | catalytic+catalytic |
|  | p.A345T/C.1315+6T>A | ?+null | 1 | 0 | 0 | 0 | catalytic+IVS |
|  | p.S349A/C.442-1G>A | ?+null | 0 | 0 | 1 | 0 | catalytic+IVS |
|  | p.S349A/p.EX6-96A>G | ?+null | 0 | 2 | 0 | 0 | catalytic+catalytic |
|  | p.S349A/C.1066-11G>A | ?+null | 1 | 0 | 0 | 0 | catalytic+IVS |
|  | p.S349A/p.Y356* | ?+null | 0 | 0 | 1 | 0 | catalytic+catalytic |
|  | p.S349A/p.V399V | ?+null | 0 | 1 | 2 | 0 | catalytic+catalytic |
|  | p.S350Y/p.EX6-96A>G | ?+null | 0 | 1 | 0 | 0 | catalytic+catalytic |
|  | p.P362T/p.EX6-96A>G | ?+null | 1 | 0 | 0 | 0 | catalytic+catalytic |
|  | p.T372R/p.R176* | ?+null | 0 | 0 | 2 | 0 | catalytic+catalytic |
|  | p.Q375H/C.168+5G>C | ?+null | 1 | 0 | 0 | 0 | catalytic+IVS |
|  | p.Q375E/p.W326* | ?+null | 1 | 0 | 0 | 0 | catalytic+catalytic |
|  | p.Y387D/p.Y356* | ?+null | 0 | 0 | 1 | 0 | catalytic+catalytic |
|  | p.F392I/p.V399V | ?+null | 2 | 0 | 0 | 0 | catalytic+catalytic |
|  | p.R400K/p.Y356* | ?+null | 0 | 1 | 0 | 0 | catalytic+catalytic |
|  | p.R400K/p.T200Nfs*6 | ?+null | 0 | 0 | 1 | 0 | catalytic+catalytic |
|  | p.R400K/C.442-1G>A | ?+null | 0 | 0 | 2 | 0 | catalytic+IVS |
|  | p.R400K/p.EX6-96A>G | ?+null | 0 | 0 | 1 | 0 | catalytic+catalytic |
|  | p.R400T/p.EX6-96A>G | ?+null | 0 | 0 | 2 | 0 | catalytic+catalytic |
|  | p.I406V/C.442-1G>A | ?+null | 1 | 0 | 0 | 0 | catalytic+IVS |
|  | p.P407L/p.EX6-96A>G | ?+null | 0 | 1 | 0 | 0 | catalytic+IVS |
|  | p.T418P/C.442-1G>A | ?+null | 1 | 0 | 0 | 0 | oligomerization+IVS |
|  | p.T418P/p.R176* | ?+null | 0 | 1 | 0 | 0 | oligomerization+catalytic |
|  | p.T418P/p.EX6-96A>G | ?+null | 1 | 0 | 0 | 0 | oligomerization+catalytic |
|  | p.T418P/p.Y325* | ?+null | 1 | 0 | 0 | 0 | oligomerization+catalytic |
|  | p.T418P/p.V399V | ?+null | 0 | 1 | 0 | 0 | oligomerization+catalytic |
|  | p.I421T/p.EX6-96A>G | ?+null | 0 | 1 | 0 | 0 | oligomerization+catalytic |
|  | p.L430P/p.Y414* | ?+null | 0 | 0 | 1 | 0 | oligomerization+oligomerization |
|  | p.L430P/p.S70del | ?+null | 0 | 1 | 0 | 0 | oligomerization+regulatory |
|  | p.T278I/p.R243Q | 1+14 | 0 | 0 | 1 | 0 | catalytic+catalytic |
|  | p.G257V/p.R243Q | 1+14 | 0 | 0 | 1 | 0 | catalytic+catalytic |
|  | p.G257V/p.R241C | 1+25 | 0 | 1 | 0 | 0 | catalytic+catalytic |
|  | p.T278I/p.R261Q | 1+44 | 0 | 1 | 0 | 0 | catalytic+catalytic |
|  | p.G257V/p.R408Q | 1+46 | 0 | 2 | 0 | 0 | catalytic+catalytic |
|  | p.G257V/p.L98V | 1+? | 0 | 0 | 1 | 0 | catalytic+regulatory |
|  | p.G257V/p.G247R | 1+? | 0 | 1 | 0 | 0 | catalytic+catalytic |
|  | p.T278I/p.S359L | 1+? | 0 | 0 | 2 | 0 | catalytic+catalytic |
|  | p.E280K/p.G247V | 2+4 | 0 | 0 | 1 | 0 | catalytic+catalytic |
|  | p.R408W/p.G247V | 2+4 | 0 | 0 | 1 | 0 | catalytic+catalytic |
|  | p.R158W/p.R243Q | 2+14 | 0 | 1 | 0 | 0 | catalytic+catalytic |
|  | p.L255S/p.R243Q | 14+2 | 0 | 1 | 0 | 0 | catalytic+catalytic |
|  | p.E280K/p.R243Q | 2+14 | 0 | 0 | 1 | 0 | catalytic+catalytic |
|  | p.P281L/p.R243Q | 2+14 | 0 | 1 | 1 | 0 | catalytic+catalytic |
|  | p.R408W/p.R243Q | 2+14 | 0 | 0 | 4 | 0 | catalytic+catalytic |
|  | p.R158W/p.R241C | 2+25 | 0 | 1 | 0 | 0 | catalytic+catalytic |
|  | p.R408W/p.V388M | 2+28 | 0 | 0 | 1 | 0 | catalytic+catalytic |
|  | p.R408W/p.I65T | 2+33 | 0 | 0 | 1 | 0 | catalytic+regulatory |
|  | p.L255S/p.R413P | 2+35 | 0 | 0 | 1 | 0 | catalytic+oligomerization |
|  | p.R408W/p.R413P | 2+35 | 0 | 0 | 1 | 0 | catalytic+oligomerization |
|  | p.E280K/p.R261Q | 2+44 | 0 | 0 | 1 | 0 | catalytic+catalytic |
|  | p.R408W/p.R408Q | 2+46 | 0 | 1 | 0 | 0 | catalytic+catalytic |
|  | p.R158W/p.A156P | 2+? | 0 | 0 | 1 | 0 | catalytic+catalytic |
|  | p.R158W/p.F260I | 2+? | 0 | 0 | 1 | 0 | catalytic+catalytic |
|  | p.L255S/p.R400K | 2+? | 0 | 1 | 0 | 0 | catalytic+catalytic |
|  | p.P281L/p.A345T | 2+? | 0 | 1 | 0 | 0 | catalytic+catalytic |
|  | p.R408W/p.L98V | 2+? | 0 | 1 | 0 | 0 | catalytic+regulatory |
|  | p.R408W/p.T372R | 2+? | 0 | 0 | 1 | 0 | catalytic+catalytic |
|  | p.R408W/p.G346R | 2+? | 0 | 0 | 1 | 0 | catalytic+catalytic |
|  | p.A434D/p.G247V | 3+4 | 1 | 0 | 0 | 0 | oligomerization+catalytic |
|  | p.R252Q/p.R243Q | 3+14 | 0 | 1 | 3 | 0 | catalytic+catalytic |
|  | p.A434D/p.R243Q | 3+14 | 2 | 6 | 3 | 0 | oligomerization+catalytic |
|  | p.R252Q/p.R241C | 3+25 | 0 | 1 | 0 | 0 | catalytic+catalytic |
|  | p.A434D/p.R241C | 3+25 | 1 | 0 | 0 | 0 | oligomerization+catalytic |
|  | p.A434D/p.R241H | 3+25 | 1 | 0 | 0 | 0 | oligomerization+catalytic |
|  | p.R252Q/p.R413P | 3+35 | 0 | 0 | 1 | 0 | catalytic+oligomerization |
|  | p.A434D/p.R413P | 3+35 | 0 | 2 | 0 | 0 | oligomerization+oligomerization |
|  | p.R252Q/p.R261Q | 3+44 | 0 | 0 | 0 | 1 | catalytic+catalytic |
|  | p.A434D/p.R261Q | 3+44 | 1 | 1 | 0 | 0 | oligomerization+catalytic |
|  | p.A434D/p.V230I | 3+63 | 1 | 0 | 0 | 0 | oligomerization+catalytic |
|  | p.R252Q/p.Q419R | 3+71 | 1 | 0 | 0 | 0 | catalytic+oligomerization |
|  | p.R252Q/p.R53H | 3+79 | 1 | 0 | 0 | 0 | catalytic+regulatory |
|  | p.R252Q/p.G344D | 3+? | 0 | 0 | 1 | 0 | catalytic+catalytic |
|  | p.A434D/p.F233L | 3+? | 0 | 1 | 0 | 0 | oligomerization+catalytic |
|  | p.A434D/p.T418P | 3+? | 0 | 1 | 0 | 0 | oligomerization+oligomerization |
|  | p.A434D/p.R400K | 3+? | 0 | 1 | 0 | 0 | oligomerization+catalytic |
|  | p.G247V/p.R158Q | 10+4 | 0 | 0 | 1 | 0 | catalytic+catalytic |
|  | p.G247V/p.R243Q | 4+14 | 0 | 2 | 3 | 0 | catalytic+catalytic |
|  | p.G247V/p.R241C | 4+25 | 1 | 0 | 0 | 0 | catalytic+catalytic |
|  | p.G247V/p.R261Q | 4+44 | 0 | 1 | 0 | 0 | catalytic+catalytic |
|  | p.G247V/p.R408Q | 4+46 | 0 | 1 | 0 | 0 | catalytic+catalytic |
|  | p.G247V/p.F233L | 4+? | 0 | 1 | 0 | 0 | catalytic+catalytic |
|  | p.G247V/p.G247R | 4+? | 0 | 0 | 0 | 1 | catalytic+catalytic |
|  | p.G247V/p.R400K | 4+? | 0 | 1 | 0 | 0 | catalytic+catalytic |
|  | p.A259T/p.P314T | 6+? | 1 | 0 | 0 | 0 | catalytic+catalytic |
|  | p.F161S/p.R252W | 7+0 | 0 | 0 | 1 | 0 | catalytic+catalytic |
|  | p.F161S/p.R243Q | 7+14 | 0 | 0 | 2 | 0 | catalytic+catalytic |
|  | p.F161S/p.R241C | 7+25 | 1 | 0 | 0 | 0 | catalytic+catalytic |
|  | p.G312V/p.R241C | 7+25 | 2 | 0 | 0 | 0 | catalytic+catalytic |
|  | p.F161S/p.R413P | 7+35 | 0 | 0 | 2 | 0 | catalytic+oligomerization |
|  | p.G312V/p.R413P | 7+35 | 0 | 0 | 1 | 0 | catalytic+oligomerization |
|  | p.L255V/p.F392I | 7+? | 1 | 0 | 0 | 0 | catalytic+catalytic |
|  | p.G312V/p.T372S | 7+? | 1 | 0 | 0 | 0 | catalytic+catalytic |
|  | p.R158Q/p.R243Q | 10+14 | 0 | 1 | 0 | 0 | catalytic+catalytic |
|  | p.R158Q/p.R241C | 10+25 | 1 | 0 | 0 | 0 | catalytic+catalytic |
|  | p.R158Q/p.V388M | 10+28 | 0 | 1 | 0 | 0 | catalytic+catalytic |
|  | p.R243Q/p.R241H | 14+23 | 1 | 1 | 1 | 0 | catalytic+catalytic |
|  | p.R243Q/p.R241C | 14+25 | 13 | 15 | 1 | 0 | catalytic+catalytic |
|  | p.R243Q/p.L52S | 14+27 | 1 | 0 | 0 | 0 | catalytic+regulatory |
|  | p.R243Q/p.V388M | 14+28 | 0 | 1 | 1 | 0 | catalytic+catalytic |
|  | p.R243Q/p.I65T | 14+33 | 0 | 1 | 2 | 1 | catalytic+regulatory |
|  | p.R243Q/p.R413P | 14+35 | 0 | 4 | 24 | 1 | catalytic+oligomerization |
|  | p.R243Q/p.R261Q | 14+44 | 1 | 1 | 4 | 0 | catalytic+catalytic |
|  | p.R243Q/p.R408Q | 14+46 | 2 | 6 | 1 | 2 | catalytic+catalytic |
|  | p.R243Q/p.A373T | 14+56 | 1 | 0 | 0 | 0 | catalytic+catalytic |
|  | p.R243Q/p.V230I | 14+63 | 4 | 0 | 0 | 0 | catalytic+catalytic |
|  | p.R243Q/p.A403V | 14+66 | 2 | 0 | 0 | 0 | catalytic+catalytic |
|  | p.R243Q/p.Q419R | 14+71 | 4 | 0 | 0 | 0 | catalytic+oligomerization |
|  | p.R243Q/p.D415N | 14+72 | 1 | 0 | 0 | 0 | catalytic+oligomerization |
|  | p.R243Q/p.R53H | 14+79 | 6 | 0 | 0 | 0 | catalytic+regulatory |
|  | p.R243Q/p.A47E | 14+? | 0 | 2 | 0 | 0 | catalytic+regulatory |
|  | p.R243Q/p.E56D | 14+? | 0 | 0 | 1 | 0 | catalytic+regulatory |
|  | p.R243Q/p.H107R | 14+? | 2 | 0 | 0 | 0 | catalytic+regulatory |
|  | p.R243Q/p.G148R | 14+? | 0 | 1 | 0 | 0 | catalytic+catalytic |
|  | p.R243Q/p.R169G | 14+? | 0 | 1 | 0 | 0 | catalytic+catalytic |
|  | p.R243Q/p.R169C | 14+? | 1 | 0 | 0 | 0 | catalytic+catalytic |
|  | p.R243Q/p.Q172H | 14+? | 1 | 0 | 0 | 0 | catalytic+catalytic |
|  | p.R243Q/p.G188D | 14+? | 0 | 0 | 1 | 0 | catalytic+catalytic |
|  | p.R243Q/p.P211S | 14+? | 1 | 0 | 0 | 0 | catalytic+catalytic |
|  | p.R243Q/p.I224T | 14+? | 0 | 0 | 1 | 0 | catalytic+catalytic |
|  | p.R243Q/p.S231P | 14+? | 0 | 0 | 0 | 1 | catalytic+catalytic |
|  | p.R243Q/p.G239A | 14+? | 0 | 0 | 1 | 0 | catalytic+catalytic |
|  | p.R243Q/p.G247R | 14+? | 0 | 3 | 1 | 0 | catalytic+catalytic |
|  | p.R243Q/p.P275L | 14+? | 0 | 0 | 1 | 0 | catalytic+catalytic |
|  | p.R243Q/p.M276K | 14+? | 1 | 0 | 0 | 0 | catalytic+catalytic |
|  | p.R243Q/p.P292L | 14+? | 0 | 0 | 1 | 0 | catalytic+catalytic |
|  | p.R243Q/p.A322D | 14+? | 1 | 0 | 0 | 0 | catalytic+catalytic |
|  | p.R243Q/p.I324N | 14+? | 0 | 1 | 2 | 0 | catalytic+catalytic |
|  | p.R243Q/p.A345T | 14+? | 0 | 1 | 3 | 0 | catalytic+catalytic |
|  | p.R243Q/p.S349A | 14+? | 0 | 1 | 1 | 0 | catalytic+catalytic |
|  | p.R243Q/p.F392I | 14+? | 1 | 0 | 0 | 0 | catalytic+catalytic |
|  | p.R243Q/p.R400T | 14+? | 0 | 1 | 2 | 0 | catalytic+catalytic |
|  | p.R243Q/p.R400K | 14+? | 0 | 0 | 1 | 0 | catalytic+catalytic |
|  | p.R243Q/p.T418P | 14+? | 0 | 1 | 0 | 0 | catalytic+oligomerization |
|  | p.R241H/p.R155H | 23+44 | 1 | 0 | 0 | 0 | catalytic+catalytic |
|  | p.R241C/p.I65T | 25+33 | 0 | 1 | 1 | 0 | catalytic+regulatory |
|  | p.R241C/p.R413P | 25+35 | 1 | 2 | 1 | 0 | catalytic+oligomerization |
|  | p.R241C/p.R261Q | 25+44 | 1 | 0 | 0 | 0 | catalytic+catalytic |
|  | p.R241C/p.R408Q | 25+46 | 4 | 0 | 0 | 0 | catalytic+catalytic |
|  | p.R241C/p.R53H | 25+79 | 0 | 1 | 0 | 0 | catalytic+regulatory |
|  | p.R241C/p.A156P | 25+? | 1 | 1 | 0 | 0 | catalytic+catalytic |
|  | p.R241C/p.R169S | 25+? | 1 | 0 | 0 | 0 | catalytic+catalytic |
|  | p.R241C/p.Y206C | 25+? | 0 | 1 | 0 | 0 | catalytic+catalytic |
|  | p.R241C/p.E214G | 25+? | 1 | 0 | 0 | 0 | catalytic+catalytic |
|  | p.R241C/p.H220P | 25+? | 2 | 0 | 0 | 0 | catalytic+catalytic |
|  | p.R241C/p.G239D | 25+? | 0 | 2 | 0 | 0 | catalytic+catalytic |
|  | p.R241C/p.G247R | 25+? | 1 | 0 | 0 | 0 | catalytic+catalytic |
|  | p.R241C/p.S349A | 25+? | 1 | 1 | 0 | 0 | catalytic+catalytic |
|  | p.R241C/p.F392I | 25+? | 1 | 0 | 0 | 0 | catalytic+catalytic |
|  | p.R241C/p.I421T | 25+? | 1 | 0 | 0 | 0 | catalytic+oligomerization |
|  | p.R241C/p.L242F | 25+? | 1 | 0 | 0 | 0 | catalytic+catalytic |
|  | p.R241C/p.L430P | 25+? | 1 | 0 | 0 | 0 | catalytic+oligomerization |
|  | p.V388M/p.L308F | 28+49 | 1 | 0 | 0 | 0 | catalytic+catalytic |
|  | p.V388M/p.R53H | 28+79 | 0 | 0 | 1 | 0 | catalytic+regulatory |
|  | p.V388M/p.I421T | 28+? | 1 | 1 | 0 | 0 | catalytic+oligomerization |
|  | p.I65T/p.E56D | 33+? | 0 | 0 | 1 | 0 | regulatory+regulatory |
|  | p.I65T/p.T418P | 33+? | 0 | 1 | 0 | 0 | regulatory+oligomerization |
|  | p.R413P/p.R261Q | 35+44 | 0 | 0 | 1 | 0 | oligomerization+catalytic |
|  | p.R413P/p.R408Q | 35+46 | 0 | 1 | 1 | 0 | oligomerization+catalytic |
|  | p.R413P/p.Q419R | 35+71 | 1 | 0 | 0 | 0 | oligomerization+oligomerization |
|  | p.R413P/p.R53H | 35+79 | 1 | 0 | 0 | 0 | oligomerization+regulatory |
|  | p.R413P/p.D75V | 35+? | 0 | 0 | 1 | 0 | oligomerization+regulatory |
|  | p.R413P/p.R157K | 35+? | 0 | 0 | 1 | 0 | oligomerization+catalytic |
|  | p.R413P/p.R169S | 35+? | 1 | 0 | 0 | 0 | oligomerization+catalytic |
|  | p.R413P/p.H170R | 35+? | 0 | 0 | 1 | 0 | oligomerization+catalytic |
|  | p.R413P/p.P175S | 35+? | 0 | 0 | 1 | 0 | oligomerization+catalytic |
|  | p.R413P/p.G247R | 35+? | 0 | 0 | 1 | 0 | oligomerization+catalytic |
|  | p.R413P/p.M276K | 35+? | 1 | 0 | 0 | 0 | oligomerization+catalytic |
|  | p.R413P/p.I324N | 35+? | 0 | 0 | 1 | 0 | oligomerization+catalytic |
|  | p.R413P/p.S350Y | 35+? | 0 | 0 | 1 | 0 | oligomerization+catalytic |
|  | p.R413P/p.Y387D | 35+? | 0 | 0 | 1 | 0 | oligomerization+catalytic |
|  | p.R413P/p.T418P | 35+? | 0 | 1 | 0 | 0 | oligomerization+oligomerization |
|  | p.R413P/p.Q375E | 35+? | 1 | 0 | 0 | 0 | oligomerization+catalytic |
|  | p.L48S/p.G247R | 39+? | 0 | 1 | 0 | 0 | regulatory+catalytic |
|  | p.R261Q/p.E56D | 44+? | 0 | 1 | 0 | 0 | catalytic+regulatory |
|  | p.R261Q/p.A156P | 44+? | 0 | 0 | 1 | 0 | catalytic+catalytic |
|  | p.R261Q/p.S349A | 44+? | 0 | 1 | 0 | 0 | catalytic+catalytic |
|  | p.R261Q/p.Y387D | 44+? | 0 | 1 | 0 | 0 | catalytic+catalytic |
|  | p.R408Q/p.R169C | 46+? | 1 | 0 | 0 | 0 | catalytic+catalytic |
|  | p.R408Q/p.R169S | 46+? | 1 | 0 | 0 | 0 | catalytic+catalytic |
|  | p.R408Q/p.G148V | 46+? | 1 | 0 | 0 | 0 | catalytic+catalytic |
|  | p.R408Q/p.T418P | 46+? | 1 | 0 | 0 | 0 | catalytic+oligomerization |
|  | p.R408Q/p.I421T | 46+? | 1 | 0 | 0 | 0 | catalytic+oligomerization |
|  | p.V230I/p.R53H | 63+79 | 0 | 1 | 0 | 0 | catalytic+regulatory |
|  | p.V230I/p.I224T | 63+? | 1 | 0 | 0 | 0 | catalytic+catalytic |
|  | p.Q419R/p.G344S | 71+? | 1 | 0 | 0 | 0 | oligomerization+catalytic |
|  | p.R53H/p.C265R | 79+? | 1 | 0 | 0 | 0 | regulatory+catalytic |
|  | p.R53H/p.R400K | 79+? | 2 | 0 | 0 | 0 | regulatory+catalytic |
|  | p.R53H/p.T418P | 79+? | 1 | 0 | 0 | 0 | regulatory+oligomerization |
|  | p.E56D/p.A156P | ?+? | 0 | 0 | 1 | 0 | regulatory+catalytic |
|  | p.E56D/p.M276K | ?+? | 0 | 1 | 0 | 0 | regulatory+catalytic |
|  | p.E56D/p.P292S | ?+? | 0 | 0 | 1 | 0 | regulatory+catalytic |
|  | p.H107R/p.H170R | ?+? | 1 | 0 | 0 | 0 | catalytic+catalytic |
|  | p.Y154H/p.F392I | ?+? | 1 | 0 | 0 | 0 | catalytic+catalytic |
|  | p.A156P/p.R400T | ?+? | 0 | 0 | 1 | 0 | catalytic+catalytic |
|  | p.A156P/p.T418P | ?+? | 0 | 1 | 0 | 0 | catalytic+oligomerization |
|  | p.A156P/p.H220P | ?+? | 0 | 0 | 1 | 0 | catalytic+catalytic |
|  | p.H170P/p.D296H | ?+? | 1 | 0 | 0 | 0 | catalytic+catalytic |
|  | p.F233L/p.S349A | ?+? | 1 | 0 | 0 | 0 | catalytic+catalytic |
|  | p.G247R/p.A345T | ?+? | 0 | 0 | 0 | 1 | catalytic+catalytic |
|  | p.G247R/p.P362T | ?+? | 0 | 1 | 0 | 0 | catalytic+catalytic |
|  | p.G247R/p.F331S | ?+? | 1 | 0 | 0 | 0 | catalytic+catalytic |
|  | p.Q267L/p.M276K | ?+? | 0 | 0 | 0 | 1 | catalytic+catalytic |
|  | p.H271R/p.S349A | ?+? | 0 | 0 | 1 | 0 | catalytic+catalytic |
|  | p.P314T/p.R400K | ?+? | 1 | 0 | 0 | 0 | catalytic+catalytic |
|  | p.A345T/p.D415Y | ?+? | 0 | 0 | 1 | 0 | catalytic+oligomerization |
|  | p.L367V/p.F392I | ?+? | 1 | 0 | 0 | 0 | catalytic+catalytic |
